# Two new species of *Rhipidoglossum* (Orchidaceae, Angraecinae) from Central Africa, probably pollinated by settling moths

**DOI:** 10.3897/phytokeys.274.184429

**Published:** 2026-04-24

**Authors:** Arthur Macedo, Vincent Droissart, Štěpán Janeček, Yannick Klomberg, Marcelo Trovó, Tariq Stévart, João Farminhão

**Affiliations:** 1 Jardim Botânico da Universidade de Coimbra, Calçada Martim de Freitas, Coimbra, Portugal Botanic Garden Meise Meise Belgium; 2 Centre for Functional Ecology, Laboratório Associado TERRA, Departamento de Ciências da Vida, Universidade de Coimbra, Coimbra, Portugal Université Libre de Bruxelles Brussels Belgium; 3 Herbarium et Bibliothèque de Botanique africaine, Université Libre de Bruxelles, Campus de la Plaine, Brussels, Belgium Charles University Prague Czech Republic; 4 AMAP Lab, Université de Montpellier, IRD, CNRS, CIRAD, INRAE, Montpellier, France Universidade Federal do Rio de Janeiro Rio de Janeiro Brazil; 5 Missouri Botanical Garden, Africa and Madagascar Department, St. Louis, MO, USA Missouri Botanical Garden St. Louis United States of America; 6 Department of Ecology, Faculty of Science, Charles University, Viničná 7, CZ-12844 Prague, Czech Republic Jardim Botânico da Universidade de Coimbra Coimbra Portugal; 7 Departamento de Botânica, Universidade Federal do Rio de Janeiro, Av. Carlos Chagas Filho 373, Cidade Universitária, Rio de Janeiro, Brazil Departamento de Ciências da Vida, Universidade de Coimbra Coimbra Portugal; 8 Botanic Garden Meise, Domein van Bouchout, Meise, Belgium Université de Montpellier Montpellier France

**Keywords:** Afromontane biodiversity, epiphytic diversity, moth pollination, Nectariniidae, orchid taxonomy, Vandeae

## Abstract

A revision of Central African specimens of *Rhipidoglossum* has revealed two new species from the Cameroon Volcanic Line (CVL) and the lowland and peripheric forests of the Guineo-Congolian rainforest block. *Rhipidoglossum acuminifolium***sp. nov**. is endemic to the CVL and is morphologically related to *R. delepierreanum* from the Western Rift Mountains. This disjunction may suggest historical connectivity or long-distance dispersal between these montane systems. Footage from Mount Cameroon recorded the settling moth *Afroracotis cf. squalida* (Lepidoptera, Erebidae) as a potential pollinator of *R. acuminifolium*, as well as numerous other plant–animal interactions, including only the second documented orchid–orthopteran interaction, involving nectaring by raspy crickets (Orthoptera, Gryllacrididae). *Rhipidoglossum acuminifolium* is preliminarily assessed here as Endangered (EN) under the IUCN Red List criteria. The morphologically allied *R. delepierreanum* is here newly recorded for Burundi, the Democratic Republic of the Congo (DRC), and Uganda. In turn, *Rhipidoglossum falcatulum***sp. nov**. is described from the Central African Republic and the DRC, where it occurs in the Congolian and Central Zambezian regions, in Guineo-Congolian lowland forests and *muhulu* vegetation. It is preliminarily assessed as Endangered (EN), and it is also hypothesised to be phalaenophilous. These two novelties increase the species diversity of *Rhipidoglossum* up to 56 species in Tropical Africa and the Gulf of Guinea Islands.

## Introduction

*Rhipidoglossum* Schltr. (Orchidaceae, Vandeae, Angraecinae) is currently the most species-rich angraecoid genus in continental Tropical Africa and the Gulf of Guinea Islands encompassing 54 accepted species, including some taxa formerly included in *Diaphananthe* Schltr. (African Plant Database 2025). It is defined by the presence of an undivided lip and a non-papillate rostellum bearing a prominent midlobe (Farminhão et al. 2018) and is part of the mystacidioid clade (Farminhão et al. 2021). The present study, focused on the description of two novelties from Central Africa, follows a series of species descriptions published over the past 15 years (Fischer et al. 2011; Farminhão 2016, 2021; Farminhão and Cribb 2020; Cribb and Hemp 2022; Macedo et al. 2025).

The highland rainforests of the Cameroon Volcanic Line (CVL) are an important diversity centre for angraecoid orchids (Droissart 2009; D’haijère et al. 2022), with two species of *Rhipidoglossum* being confined to this ecoregion: *Rhipidoglossum polyanthum* (Kraenzl.) Szlach. & Olszewski (Szlachetko and Olszewski 2021) and *Rhipidoglossum polydactylum* (Kraenzl.) Garay (Nodza et al. 2022). A revision of herbarium material of *Rhipidoglossum* from the CVL revealed what could possibly represent a third undescribed endemic species collected in the montane forests of Bioko Island, Mount Cameroon, and the Banyang Mbo Wildlife Sanctuary (in a small northern extension of the Bakossi mountains). A first non-flowering specimen was collected in 1967, in southern Bioko, by William W. Sanford (1924–2002), who determined it as an unidentified *Diaphananthe* Schltr. Probably due to this uncertainty, this material was not mentioned in the first orchid catalogue for the island (Sanford 1971). Later, this specimen, together with a 1973 collection from Mount Cameroon made by Raija Linnavuori (1942–1983), collected with the entomologist Rauno Linnavuori (Wilson et al. 2017), was tentatively identified by Phillip J. Cribb as an undescribed species close to *Diaphananthe tenuicalcar* Summerh. [=*Rhipidoglossum tenuicalcar* (Summerh.) Garay]. The species was recorded in Mount Cameroon on two other occasions: in 2007 by Jean-Michel Hervouet, who photographed a flowering plant and in 2017 as *Rhipidoglossum* sp., by two of the authors of the present article (YK, SJ), who collected specimens, and recorded flower visitors (Klomberg et al. 2022; Janeček et al. 2022, 2024: Uceda-Gómez et al. 2023). Two additional records by one of the authors (VD) are based on non-flowering plants ascribable to this new *Rhipidoglossum*: a 2008 herbarium gathering from the Sanctuary of Banyang Mbo; and plants observed *in situ*, in 2021, in the little-known Badoumkassa submontane forest near “Col de Bana” in the West Region of Cameroon. One fruiting plant from the latter locality was kept in cultivation at the Yaoundé orchid shade house, but unfortunately died without producing a specimen (cultivation number YA8047). The novelty is most similar to *Rhipidoglossum delepierreanum* (J.-P.Lebel & Geerinck) Eb.Fisch., Killmann, J.-P.Lebel and Delep., which is only known from the montane forests of western Rwanda (Fischer et al. 2011; African Plant Database 2025). Thus, a detailed morphological study was needed to clarify whether the plants from the CVL corresponded to a new species or represented a significant range extension for *R. delepierreanum*.

A second undescribed *Rhipidoglossum* was identified based on specimens from the Central African Republic (CAR) and the Democratic Republic of the Congo (DRC). The oldest specimens were collected between 1949 and 1951 by the French missionary, linguist, ethnologist and botanist Charles Tisserant (1886–1962) and his team, in the forests around Boukoko. Although these specimens are not cited in the catalogues of the orchid flora of the CAR (Tisserant 1950; Cribb and Fay 1987), they were identified as *Diaphananthe rutila* (Rchb.f.) Summerh. [=*Rhipidoglossum rutilum* (Rchb.f.) Schltr.] and *D. erectocalcarata* (De Wild.) Summerh. [=*Rhipidoglossum erectocalcaratum* (De Wild.) Summerh.] in herbaria. Later collections of the same species were made by Jan Bokdam (1946–2021) in 1973 and Michel Schaijes (1929–2017), between 1982 and 1986, in the Provinces of Tshopo (northern DRC) and Haut-Katanga (southern DRC), respectively. These specimens were also tentatively identified as *R. rutilum* in herbaria. Leaf and flower shape strongly suggest that these plants represent a new species, but a more detailed morphological comparison, including *Rhipidoglossum curvatum* (Rolfe) Garay with similar flower morphology, was lacking.

Here, we describe *Rhipidoglossum acuminifolium* sp. nov., from the CVL, and *R. falcatulum* sp. nov., from the CAR and DRC, in a taxonomic treatment including iconography, synoptic tables, a distribution map, habitat and ecology accounts, along with conservation status assessments. This is followed by a discussion of the taxonomic, biogeographical, ecological, and conservation significance of these findings.

## Material and methods

We compared the material of the two novelties with herbarium specimens, as well as additional photographic and unvouchered records of *Rhipidoglossum delepierreanum* and *R. curvatum*, the two taxa more morphologically allied to the new species. This included type material housed at B, BR, BRLU, C, COI, G, K, LISC, LISU, LWI, MA, MO, P, PO, UPS, WAG, YA, Z, ZSS, and ZT (acronyms according to Thiers 2025). In total, 20 specimens and four unvouchered photographic records of *R. delepierreanum*, available through TROPICOS (Teisher and Stimmel 2026), were examined, as well as 20 selected specimens for *R. curvatum*. Dry-preserved specimens, rehydrated in Copenhagen mix, and spirit specimens were analysed under a stereomicroscope (Zeiss Stemi SV11). The general descriptive terminology follows Beentje (2016), but for the two-dimensional shapes, follows Radford et al. (1974). Morphological information was summarised into synoptic tables, deriving from the observation of newly collected samples and taxonomic literature (Summerhayes 1937, 1968; Cribb 1989; Geerinck 1992; Szlachetko and Olszewski 2001; Fischer et al. 2010; Szlachetko et al. 2021).

Habitat and ecological data were assembled from herbarium labels and fieldwork conducted by JF in Nyungwe National Park (Rwanda, January 2018), VS in Bandoumkassa (Cameroon, West Province, November 2021), and SJ and YK in Mount Cameroon (Cameroon, Southwest Province, for the projects in Klomberg et al. (2022), Uceda-Gómes et al. (2023), and Sakhalkar et al. (2023), between 2017–2020). Following the protocol described in Stévart et al. (2020), non-flowering specimens of *R. delepierreanum* and *R. acuminifolium* sp. nov. were preserved in cultivation at the orchid garden of the research station of the University of Kaiserslautern-Landau in Huye (Rwanda), and the orchid shadehouse at Yaoundé (Cameroon), respectively, but none produced flowers and all subsequently died. Unvouchered records based only on photographs are here referenced as Suppl. materials (Suppl. materials 1, 2). The observation protocol for plant-pollinator interactions, including video capturing, is described in detail in Uceda-Gómes et al. (2023).

For the assessment of the conservation status, occurrences based on georeferenced herbarium samples and photographic records (Suppl. materials 1, 2) were imported into GeoCAT (Bachman et al. 2011) to calculate the extent of occurrence (EOO) and area of occupancy (AOO) with a 2 × 2 km cell size, as suggested by IUCN (2024). A survey using Google Earth Pro software (Google 2025) was performed to identify potential threats, such as landscape changes, infrastructural encroachment, and the preservation of buffer and border areas in conservation units to support our threat assessments. A geographic distribution map was produced using the software QGIS v.3.40 (QGIS Development Team 2024). The geographical coordinates of all records, when available in the samples, were analysed, but in order to protect these species against international biodiversity traffic, here they are not presented.

## Taxonomic treatment

### Rhipidoglossum delepierreanum

Taxon classificationPlantaeAsparagalesOrchidaceae

(J.-P.Lebel & Geerinck) Eb.Fisch., Killmann, J.-P.Lebel and Delep., Die Orchidee 62(6): 445 (2011).

F3A9596D-0B96-58DD-80A3-DB74DDF61174

Diaphananthe delepierreana J.-P.Lebel & Geerinck, Belg. J. Bot. 130: 136 (1998). Basionym.

#### Type.

**Rwanda** • **Western Province (Cyangugu)**: forêt de Nyungwe-Kibazi, alt. 1800 m, *G. Delepierre 39* (holotype BR [BR0000008809643!], isotypes BR [BR6102001375171-spirit!, BR6102016350385-spirit!]).

#### Distribution.

Uganda, Democratic Republic of the Congo (DRC), Rwanda, and Burundi, being a new country record for Uganda, the DRC and Burundi. *Rhipidoglossum delepierreanum* is found in the montane forests in the Western Rift System, between 1600–2000 m a.s.l.

#### Specimens examined.

**Uganda** • **Western Province**: Bushenyi District, S. Kasyoha-Kitomi Forest, Nzozi, 1330 m, Jun 1998, st. inf., *D.L.N. Hafashimana 0643* (K s.n.); • Rukungiri district, Kayonza, Bwindi Impenetrable Forest National Reserve, 1520 m, Aug 1998, st. inf., *D.L.N Hafashimana 0684* (K s.n.); • *ibid. loc*., ca. 1700 m (5500 ft), 4 Sep 1994, fl., *Spurrier U.16* (K s.n.); • *sine loco accurato*, cultivated by L.N. Mason, Talbot Manor, Fincham, Norfolk, Nov 1960, fl., *Burgess s.n*. (cult. num. 661) (K [KSPC8301]). **Democratic Republic of the Congo** • **Ituri**: Baniari, Irimu, 14 Oct 1948, fl., *J. de Wilde 140* (BR [BR0000006803803], P [P00388678], MO*non visum*, WAG [WAG.1134679]); • *ibid. loc*., Vallée de la Maginda, 1450 m, 19 Oct 1948, fl., *J. de Wilde 167* (BR [BR0000009930506]); • South-Kivu: Kalehe, km 110 route Kavumu-Walikale, 850 m, 13 Jul 1956, st., *A.R. Christiaensen 974* (BR [BR0000021827938]); • *ibid. loc*., km 40 route Kavumu-Walikale, 1700 m, 23 Jun 1956, fr., *A.R. Christiaensen 928* (BR [BR0000016158931]). **Rwanda** • **Western Province (Cyangugu)**: Nyamasheke district, Route Pindura-Burundi, 2000 m, 1975, st. inf., *G. Troupin 15739* (BR [BR0000006627393, BR6102009937432, spirit]); • *ibid. loc*., Piste Nyungwe, Piste Bweyeye, 1700 m, 22 Mar 2002, fl., *G. Delepierre 105* (BR [BR6102016348610, spirit]); • *ibid. loc*., Kibazi, 1800 m, Mar–Apr 1997, fl., *G. Delepierre s.n*. (BR [BR0000006803575]); • **Without locality**: cultivated in Kigali, 20 Apr 2005, fl., *G. Delepierre 150* (BR [BR6102016351412]). **Burundi** • **(Probably) Bujumbura Province**: Plaine de Buhoro, 12 April 1942, fl., *M. Arbonnier 330* (BR [BR0000009931909; BR0000021311871]); Mabaye, (river) Lua, 1650 m, 22 Jun 1969, st. inf., *J. Lewalle s.n*. (K s.n.).

#### Notes.

**Figure 1.**
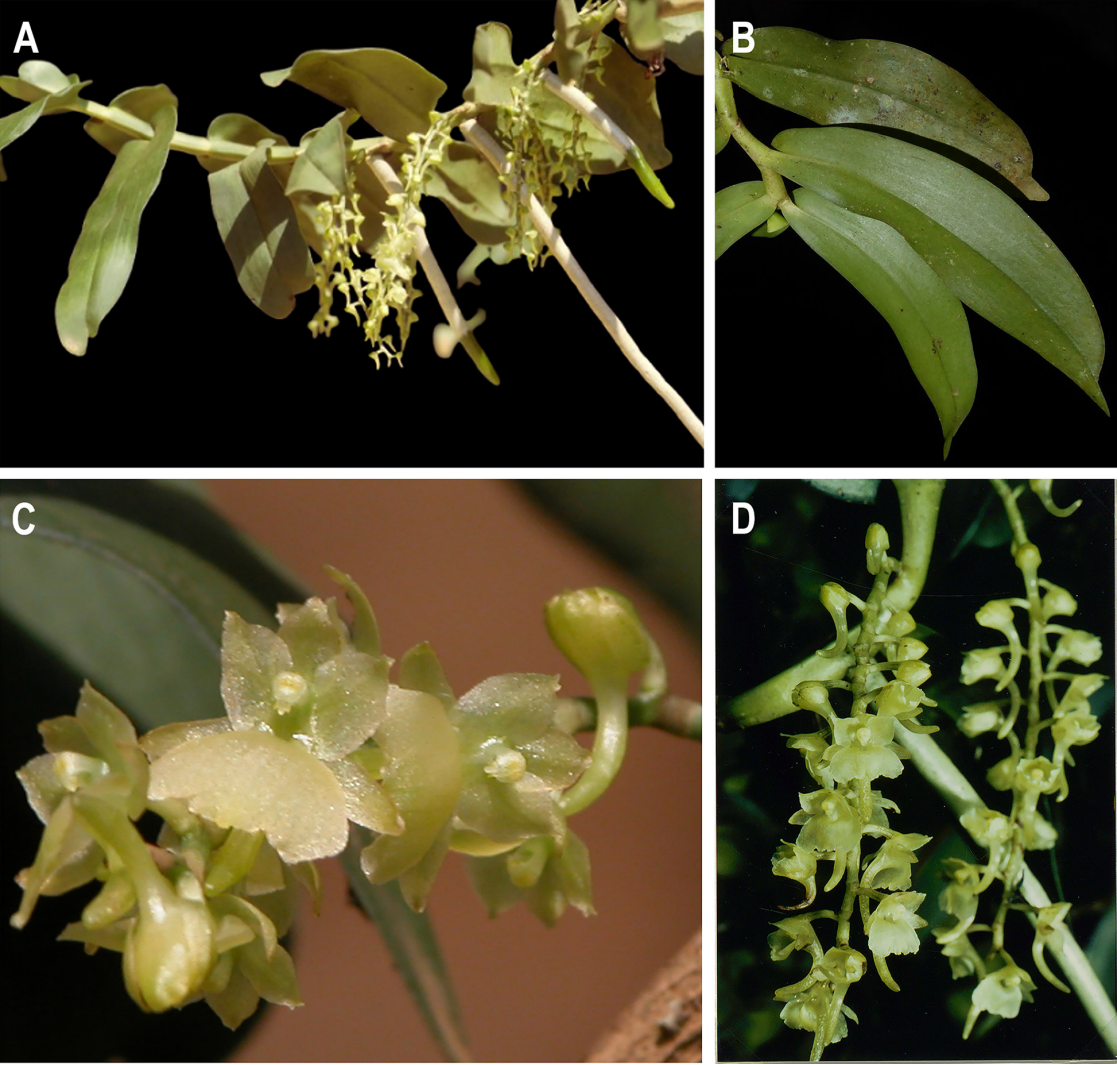
*Rhipidoglossum delepierreanum*, morphological overview. **A**. Habit; **B**. Leaves; **C, D**. Inflorescences and flower detail. **A, C**. From Rwanda, Fischer et al. (2010); **B**. Rwanda, Western Province (unvouchered record *J. Farminhão* and *B. Dumbo cult. RWA162*); **D**. First record of *R. delepierreanum* in Burundi. *M. Arbonnier 330* [BR0000009931909; BR0000021311871].

From Uganda, *R. delepierreanum* is here reported based on two non-flowering specimens previously identified in herbaria as *Rhipidoglossum bilobatum* (Summerh.) Szlach. & Olszewski; one cultivated specimen of unknown exact provenance first identified as *Diaphananthe* sp.; and one flowering specimen previously identified as *R. xanthopollinium* (Rchb.f.) Schltr. Seven specimens from the Democratic Republic of the Congo were located, including two gatherings from Ituri (*J. de Wilde 140* and *167*) and two gatherings from South-Kivu (*A. Christiaensen 928* and *974*), all originating in Eastern DRC, close to the borders with Rwanda and Uganda. They were previously identified as *Diaphananthe* sp. or *Rhipidoglossum rutilum*. Two vouchers from Burundi were located. A first gathering from Mabaye—Lua, was previously identified as *Diaphananthe aff. tenuicalcar* at K, while a second collection (*M. Arbonnier 330*, Fig. 1D), previously identified as *Diaphananthe bidens* (Afzel. ex Sw.) Schltr. at BR, lacks an exact locality, being only labelled as “plaine de Buhororo”. This locality is here interpreted as being near the village of Buhororo (Bujumbura), in northern Burundi, based on its relative proximity to Parc National de la Kibira. This area represents a plausible ecological extension of the species habitat from Nyungwe National Park (the type locality) along the same forest block.

### Rhipidoglossum acuminifolium

Taxon classificationPlantaeAsparagalesOrchidaceae

A.R.Macedo & Farminhão sp. nov.

821BBD4E-8520-58AD-AE46-27F7334423BA

urn:lsid:ipni.org:names:77379122-1

**Figure 2.**
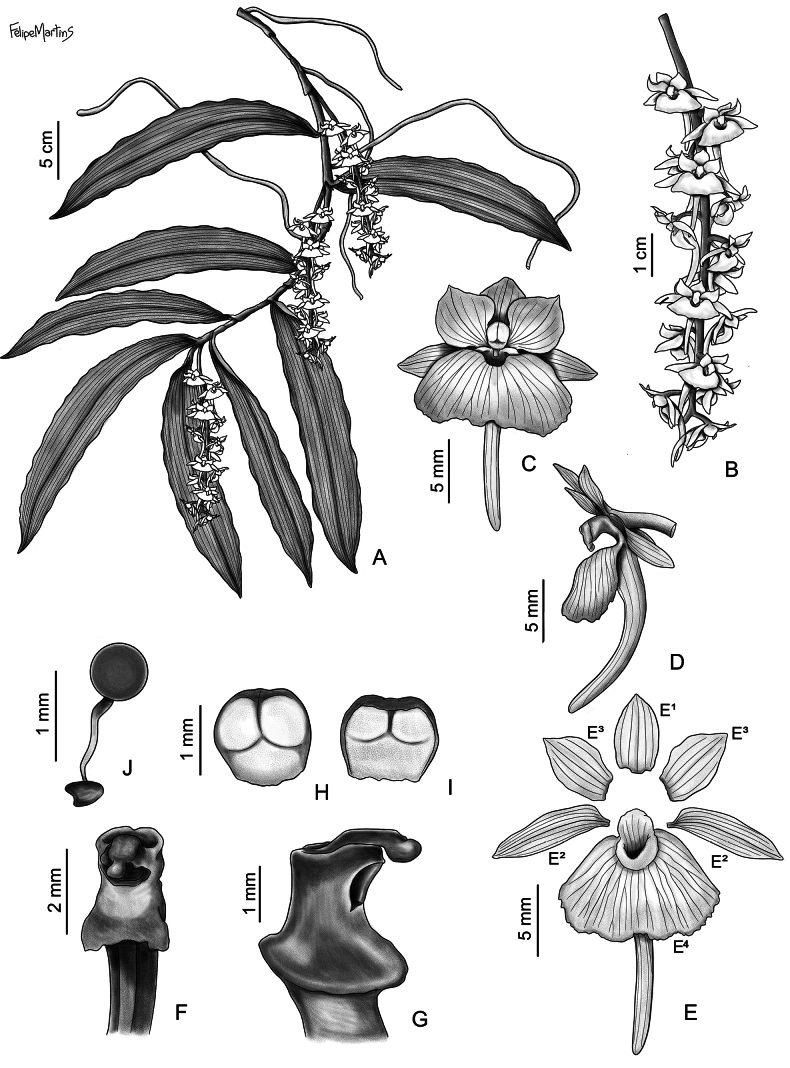
*Rhipidoglossum acuminifolium*. **A**. Habit; **B**. Inflorescence; **C, D**. Flower, frontal (**C**) and lateral (**D**) views; **E**. Flower dissected: **E^1^**. Dorsal sepal; **E^2^**. Sepals; **E^3^**. Petals; **E^4^**. Lip; **F, G**. Column, frontal (**F**) and lateral (**G**) views; **H, I**. Anther cap, adaxial (**H**) and abaxial (**I**) views; **J**. Pollinarium. Drawn from *Š. Janeček* s.n. BRLU (holotype) by Felipe Martins.

**Figure 3.**
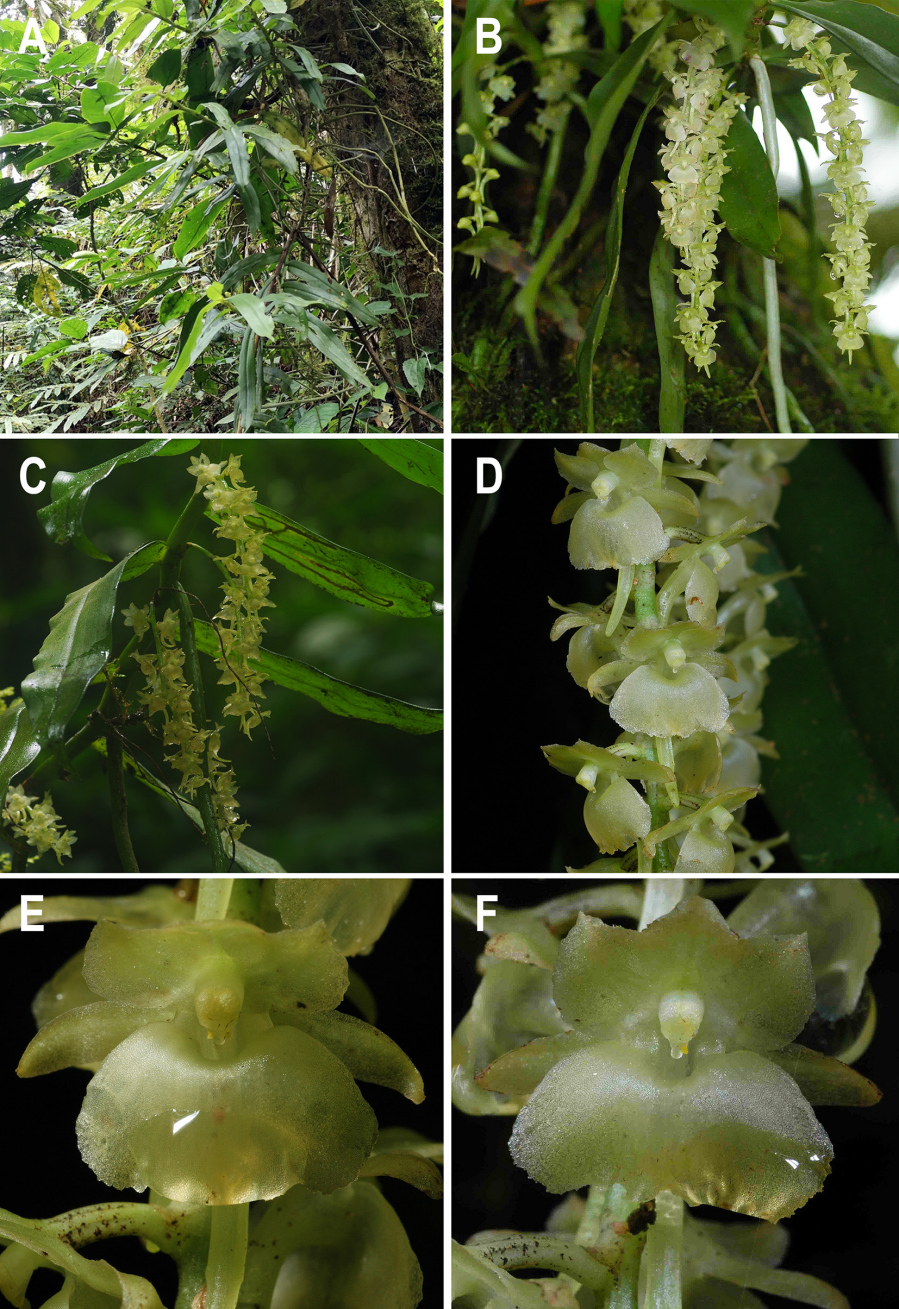
*Rhipidoglossum acuminifolium*. **A**. Habit; **B, C**. Mature inflorescences; **D**. Inflorescence close-up; **E, F**. Flowers, frontal view. Photographs by Jean-Michel Hervouet and Štěpán Janeček (**holotype***Š. Janeček* s.n. BRLU).

**Figure 4.**
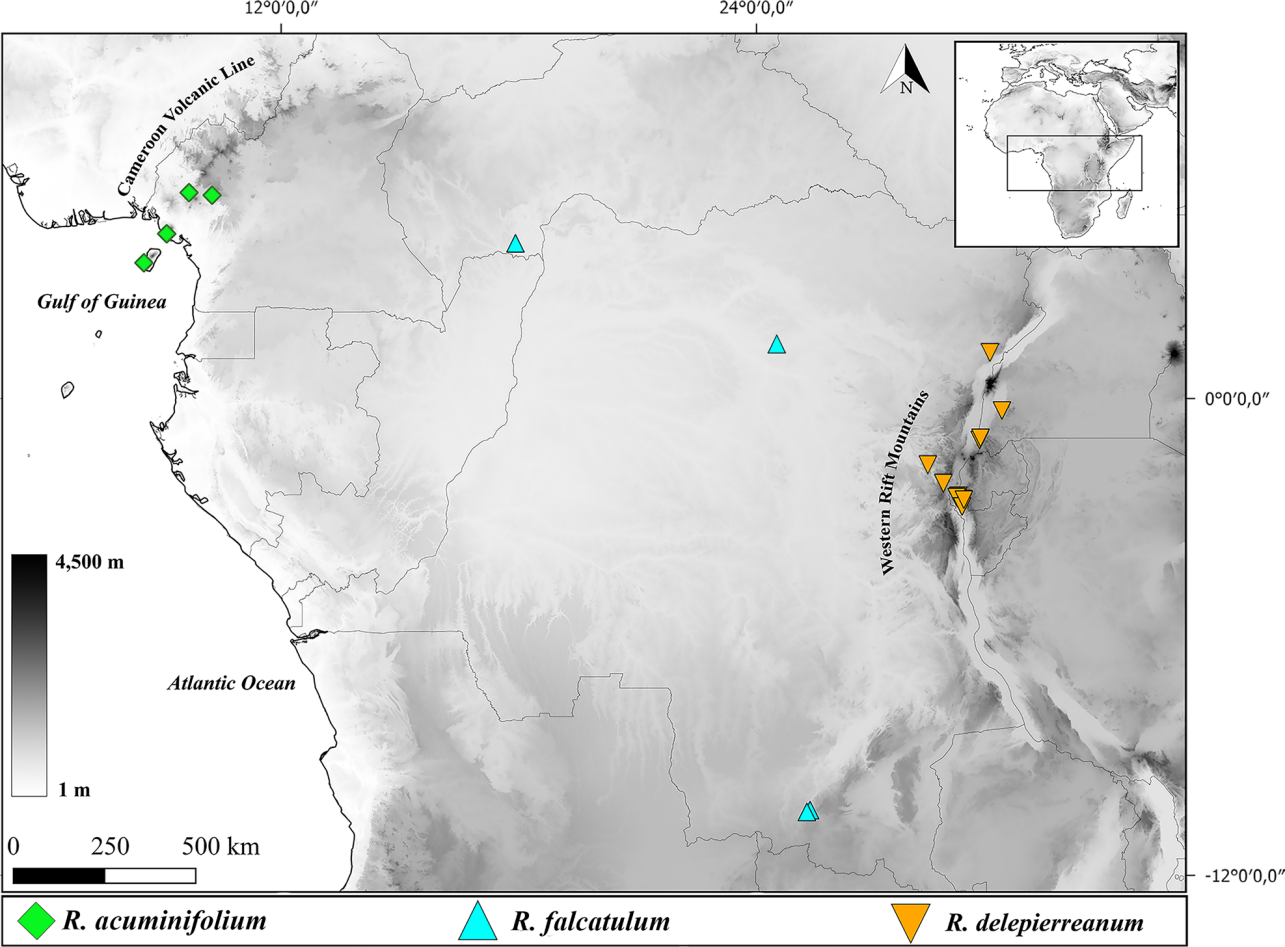
Distribution map of *Rhipidoglossum acuminifolium*, *R. falcatulum* and *R. delepierreanum* in continental Tropical Africa.

Figs 2, 3, 4, Table 1

#### Type.

**Cameroon** • **Southwest**: Fako District, Mount Cameroon, Mann’s Spring, 2150 m, 16 Aug 2019, *Š. Janeček s.n*. (holotype BRLU s.n.!, isotype YA!).

**Table 1.** Diagnostic traits of *R. acuminifolium* sp.nov. and *R. delepierreanum*, its closest morphologically related species.

	*R. acuminifolium* sp.nov.	*R. delepierreanum*
**Leaves**	narrowly elliptic to narrowly oblong, apex very unequally bilobed, one lobe acute to acuminate, 56–197 × 14–37 mm	elliptic to elliptic-oblong, apex shortly unequally bilobed, 75–10 × 20–35 mm
**Inflorescence**	17–25-flowered, up to 102 mm long	12–20-flowered, up to 130 mm long
**Dorsal sepal**	elliptic, 5–5.5 × 3–3.2 mm	elliptic, 4–4.2 × 2.5–3 mm
**Lateral sepals**	narrowly elliptic, 8–8.5 × 2.3–2.5 mm	oblong, 6–6.2 × 1.5–2 mm
**Petals**	elliptic to ovate, 5.8–6 × 3.3–3.5 mm	elliptic to widely elliptic, 4–4.2 × 3.5–4 mm
**Lip**	flabellate, slightly bilobed, lateral margin entire, apical margin incised 5.6–7.5 × 11–12 mm	flabellate, lateral margin crenulate, apically emarginate, 7–7.2 × 8–8.2 mm
**Spur**	14.2–15 × 0.2–2 mm	6–8 × 0.8–1 mm
**Column**	2.2–2.4 × 1.6–1.8 mm	1.2–1.3 × 0.8–0.9 mm
**Rostellum midlobe**	midlobe oblong with globose apex, midlobe 1.7–1.8 × 0.7 mm	midlobe oblong with globose apex, midlobe 0.4 × 0.2 mm

#### Diagnosis.

*Rhipidoglossum acuminifolium* is most similar to *Rhipidoglossum delepierreanum*, but can be distinguished by the following characteristics: leaf shape (narrowly elliptic-falcate to narrowly oblong-falcate *vs*. elliptic-falcate to oblong-falcate), including the apex (very unequally bilobed, larger lobe acute to acuminate, 12–19 mm long *vs*. unequally bilobed, larger lobe 7–11 mm long), and longer spur (14.2–15 mm long *vs*. 6–8 mm long) (Table 1).

#### Description.

Epiphytic herb, pendent, up to 120 cm long. Roots slender, basal and axillary, usually one per node, greenish-whitish, ca. 300 mm × 2 mm. Stem pendent, slender, rarely branched, up to 300 × 2–5 mm, internodes 20–30 mm long. Leaves up to 15, distichous, concolorous, narrowly elliptic-falcate to narrowly oblong-falcate, entire, margin slightly undulate, apex unequally bilobed, larger lobe 12–19 mm long, acuminate, base attenuate, 56–197 × 14–37 mm. Inflorescences up to 7, dense, 1(–2) per node, axillary, pendent, shorter than leaves, 17–25-flowered, 40–102 mm long; peduncle glabrous, 3–6 mm long; rachis glabrous, green, 37–96 mm long; bracts ochreate, whitish, 1.5 × 2 mm. Flowers whitish to pale green, pale orange during late anthesis. Pedicel and ovary cylindrical, scurfy, with dark brown scales, 5–6 × 1–1.3 mm; dorsal sepal elliptic, apex acute, base obtuse, entire, 5–5.5 × 3–3.2 mm, lateral sepal narrowly elliptic, apex and base attenuate, entire, 8–8.5 × 2.3–2.5 mm; petals elliptic to ovate, apex acute to slightly acuminate, base obtuse, entire, 5.8–6 × 3.3–3.5 mm, lip flabellate, apex bilobed, lateral margin crenulate, apical margin incised, convex, ecallose, 5.6–7.5 × 11–12 mm; spur cylindrical, acute at apex, curved, whitish to pale green 14.2–15 × 0.2–2 mm; column whitish to pale green, 2.2–2.4 mm long; anther cap galeate, frontal margin slightly undulate, whitish, 1.7–1.8 × 1.7–1.8 mm, stipites two, obclavate, translucid, viscidia two, depressed ovate, yellowish; pollinia two, orbicular, yellowish, 0.7 × 0.7 mm; rostellum trilobed, lateral lobes reduced, subtriangular, midlobe oblong with a globose apex, 1.7–1.8 × 0.7 mm. Fruit a capsule, elliptic, ribbed, 15.5–17.9 × 6.3–6.5 mm.

#### Distribution.

Equatorial Guinea (Bioko) and Cameroon (Fig. 4). *Rhipidoglossum acuminifolium* occurs in submontane to montane forest patches along the Cameroon Volcanic Line, between 550–2165 m a.s.l.

#### Habitat and ecology.

**Figure 5.**
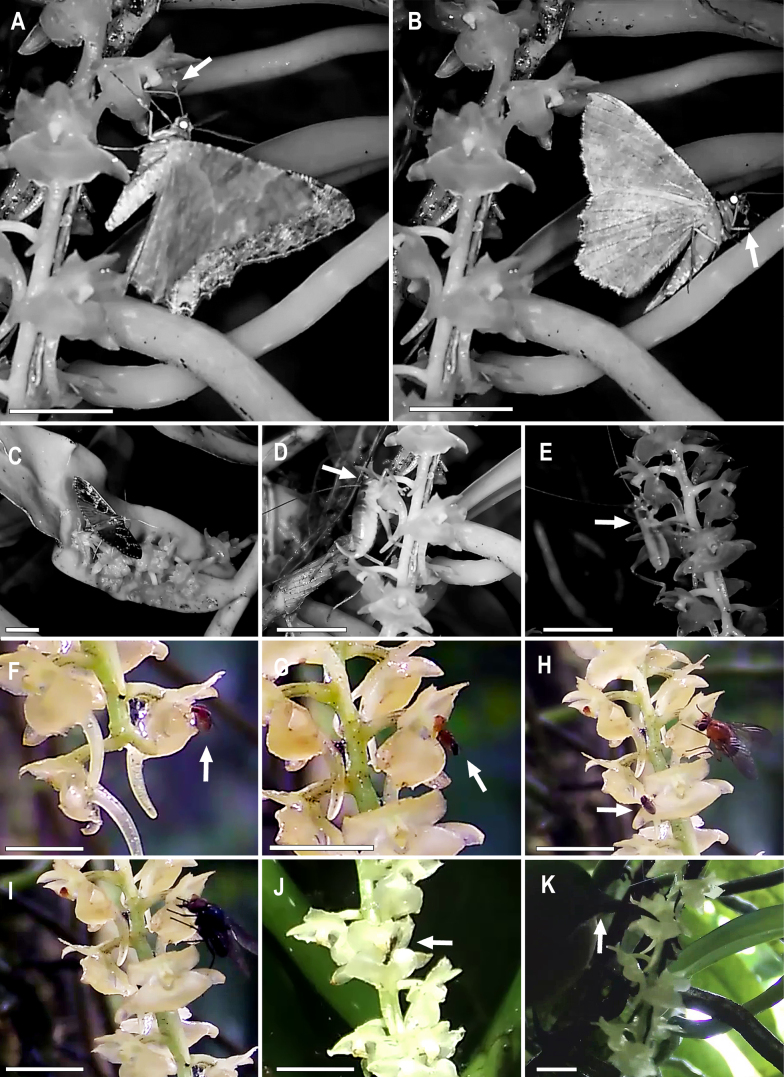
Records of floral visitors, including potential pollinators of *Rhipidoglossum acuminifolium*. **A–K**. Best frames captured from the footage recorded at Mann’s Spring, Mount Cameroon, Cameroon. **A, B**. *Afroracotis cf. squalida* (Lepidoptera, Erebidae), 11 Aug 2017, at 22:12, visiting the flowers of *R. acuminifolium*, successfully removing the pollinia (**A**) and carrying it away on the proboscis (**B**); **C**. *Paschiodes* sp. (Lepidoptera, Erebidae), 19 Aug 2017, at 23:43, visiting a second inflorescence; overview; **D, E**. Adult female and nymph of unidentified species of raspy cricket, cf. *Glomeremus* sp. (Orthoptera, Gryllacrididae, Ametroidini), 11 Aug 2017, at 19:15, visiting the flowers and collecting residues of nectar from the spur entrance; **F**. *Zaprionus* sp. (Diptera, Drosophilidae), 11 Aug 2017, at 10:48, visiting the flowers and collecting residues of nectar from the spur entrance; **G**. *Drosophila* or *Scaptodrosophila* sp. (Diptera, Drosophilidae), on 11 Aug 2017, at 11:27, visiting the flowers and collecting residues of nectar from the spur entrance and lip surface; **H**. An unidentified morphotype of fly described as “orange-whitish-dark fly” (Diptera), on 11 Aug 2017, at 12:18, visiting the flowers, collecting residues of nectar from the spur entrance; **I**. An unidentified species of *Calyptratae* (Schizophora, Diptera), on 11 Aug 2017, at 10:53, visiting the flowers, collecting residues of nectar from the spur entrance; **J**. Unidentified species of wasp (Hymenoptera), on 11 Aug 2017, at 11:41, visiting the flowers; **K**. *Cinnyris reichenowi* (Aves, Nectariniidae), on 11 Aug 2017, at 13:32, visiting and robbing nectar from the flowers. Scale bars: 1 cm. The complete record is available in Suppl. materials 3–11.

*Rhipidoglossum acuminifolium* is a trunk epiphyte, occasionally growing on lianas, occurring in primary and secondary montane and submontane forests, in partly shaded areas, at 2–5 meters above the ground, often forming large clumps hanging from the phorophyte (see Suppl. material 2). In the studies on the plant–pollinator interactions on Mount Cameroon, the first flower visitor records of *R. acuminifolium* were identified (Klomberg et al. 2022; Sakhalkar et al. 2023; Uceda-Gómes et al. 2023) (Fig. 5). The moth *Afroracotis cf. squalida* (Lepidoptera, Erebidae) successfully removed the pollinia from the column and carried them away dorsally attached to the proximal (basal) part of the proboscis (Fig. 5A, B). Another settling moth species, *Paschiodes* sp. (Lepidoptera, Erebidae), was observed visiting the flowers (Fig. 5C). In addition, insect visitors included an unidentified ametroidine raspy cricket, cf. *Glomeremus* sp. (Orthoptera, Gryllacrididae, Ametroidini) (Fig. 5D, E), fruit flies of the genera *Zaprionus* Coquillett, 1902 (Fig. 5F), *Drosophila* Fallén, 1823 and or *Scaptodrosophila* Fallén, 1823 (Diptera, Drosophilidae) (Fig. 5G), and two unidentified species of calyptrate muscoid flies (Diptera, Calyptratae) (Fig. 5H, I). The records also include an unidentified parasitoid wasp (Hymenoptera) (Fig. 5J) (Klomberg et al. 2022). The only bird visitor recorded was the Northern Double-collared Sunbird (*Cinnyris reichenowi* Sharpe, 1891) (Fig. 5K), which was observed feeding on nectar obtained directly from the spur (Fig. 5; Suppl. materials 3–11). This species is unlikely to play a role in the effective pollination of *R. acuminifolium*, acting instead as a nectar thief. In contrast, settling moths are considered the most likely primary pollinator group, owing to pollinia removal and their frequent contact with the column (Klomberg et al. 2022). *Rhipidoglossum acuminifolium* has been recorded in association with a diverse assemblage of orchid species, such as *Aerangis gravenreuthii* (Kraenzl.) Schltr., *Angraecopsis tridens* (Lindl.) Schltr., *Bulbophyllum deshmukhii* U.B.Deshmukh & J.M.H.Shaw, *Polystachya cooperi* Summerh., and *Rhipidoglossum confusum* (P.J.Cribb) Farminhão and Stévart. (African Plant Database 2025; GBIF 2025; V. Droissart pers. obs.).

#### Phenology.

Flowers in the wet season from June to late August, and January. Fruits observed in November.

#### Etymology.

The specific epithet acuminifolium refers to the shape of the larger lobe on the leaves of this species. This feature is the most evident trait used to differentiate it from its closest relative.

#### Preliminary IUCN conservation assessment.

*Rhipidoglossum acuminifolium* is known from six herbarium and spirit samples and one observation representing seven occurrences, the most recent made in 2021. Except for one (photographic) observation (in Bandoumkassa, Cameroon), all occurrences were made inside or associated with officially protected areas (Mount Cameroon National Park and Sanctuaire de Banyang Mbo, Cameroon; Gran Caldera de Luba Scientific Reserve, Equatorial Guinea (Bioko Island)), and they can be considered as still existing occurrences. These occurrences represent four biogeographical subpopulations. Three subpopulations occur in each of the three protected areas cited above. Four locations were identified with respect to the most serious plausible threat known to the ecosystems in the Cameroon Volcanic Line: habitat degradation due to the combined effects of deforestation for small-scale agriculture, road and urban expansion and potentially small-scale timber extraction (Solefack et al. 2012; Lézine et al. 2025). Evidence of direct human impact on the vegetation, small-scale agriculture, presence of buildings, and landscape changes is notable close to the Gran Caldera de Luba Scientific Reserve, Equatorial Guinea, as well as in the limits of the Mount Cameroon National Park, in Cameroon. The Sanctuaire de Banyang Mbo, also in Cameroon, appears to be a relatively stable conservation unit, with little evidence of landscape and vegetation changes, presence of crop species and buildings. The Bandoumkassa population is the most vulnerable, occurring outside any protected area and adjacent to human settlements, with intense evidence of urban expansion, agricultural encroachment, landscape alteration and clearance of primary and secondary forests. The extent of occurrence (EOO) is calculated as 6,775 km^2^ (falling within the limits for Vulnerable status under criterion B1), whereas its area of occupancy (AOO) is estimated at 16 km^2^ (within the limits for Endangered status under criterion B2), and the number of locations being equal to 4, within the limits for Endangered status under criterion Ba. The projected loss of the Bandoumkassa subpopulation, and contraction of its area of occupancy, is associated with a continuing decline in EOO, AOO, habitat extent and quality, and mature individuals (b i, ii, iii, iv). *Rhipidoglossum acuminifolium* is thus assigned a preliminary risk of extinction status of **Endangered: EN** B2ab(i, ii, iii, iv).

#### Paratypes.

**Cameroon** • **Southwest**: Koupé-Manengouba, Sanctuaire de Banyang Mbo, Village de Bejange, 550 m, 1 Feb 2008, st., *V. Droissart 648* (BRLU s.n.); • District Fako, Mount Cameroon, “loc. 11”, 17–18 Jun 1973, fl., *R. Linnavuori s.n*. (H [H1259726, H1210167]); • *ibid. loc*., near Mann’s spring, 2165 m, 14 Aug 2019, fl., *Š. Janeček s.n*. (BRLU s.n.). **Equatorial Guinea** • **Bioko**: Caldera [de] San Carlos to Ruiché, 11 Jan 1967, fl., *W.W. Sanford 4360* (K s.n.).

### Rhipidoglossum falcatulum

Taxon classificationPlantaeAsparagalesOrchidaceae

Farminhão & A.R. Macedo sp. nov.

55ADA4E8-B451-514C-BB52-D13B38BB1D98

urn:lsid:ipni.org:names:77379125-1

**Figure 6.**
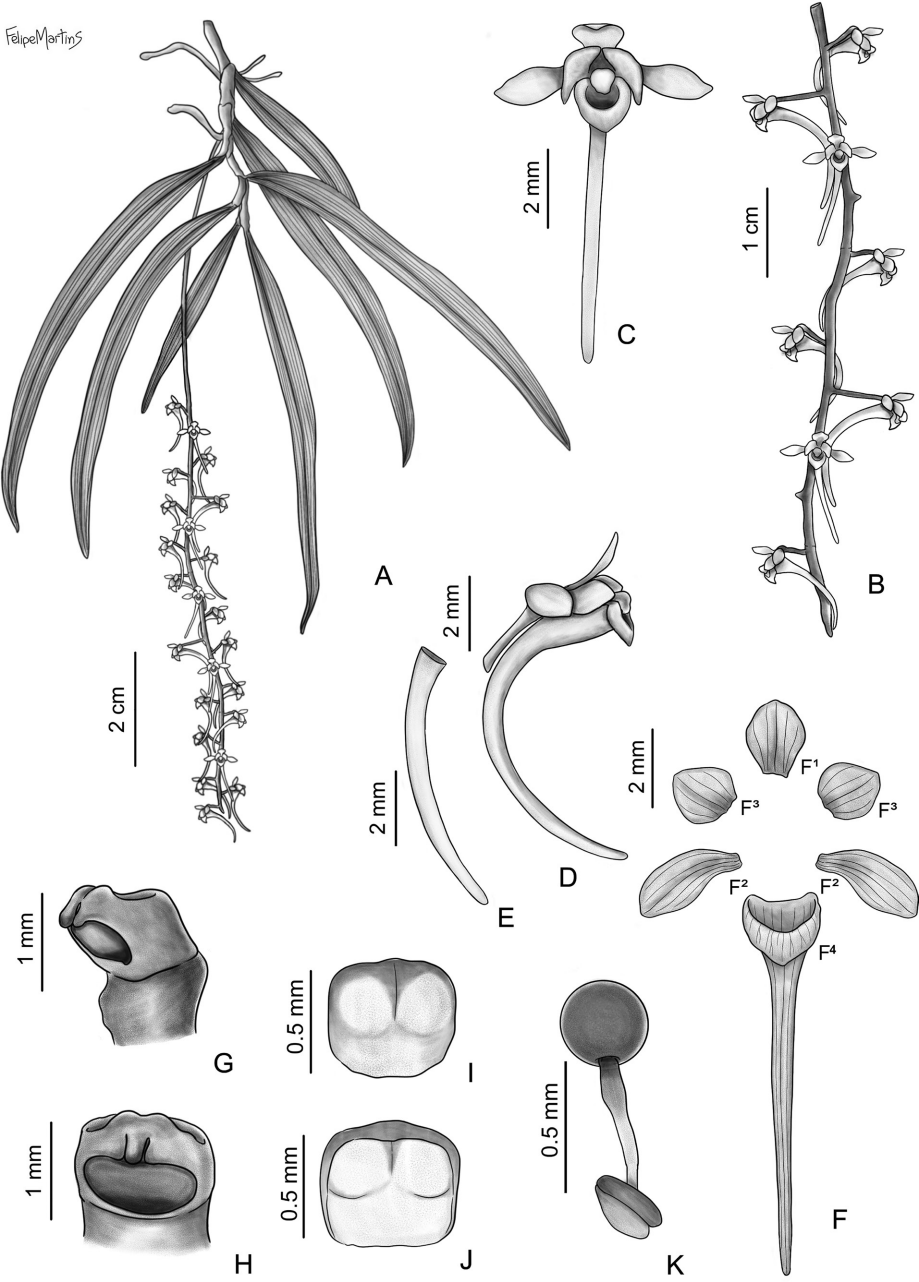
*Rhipidoglossum falcatulum*. **A**. Habit; **B**. Inflorescence; **C**. Flower, frontal view; **D, E**. Spur variability; **F**. Flower dissected: **F^1^**. Dorsal sepal; **F^2^**. Sepals; **F^3^**. Petals; **F^4^**. Lip; **G, H**. Column, frontal (**G**) and lateral (**H**) views; **I, J**. Anther cap, adaxial (**I**) and abaxial (**J**) views; **K**. Pollinarium. Drawn from *M. Schaijes 1298* [BR0000025200034, BR0000025200027] by Felipe Martins.

**Figure 7.**
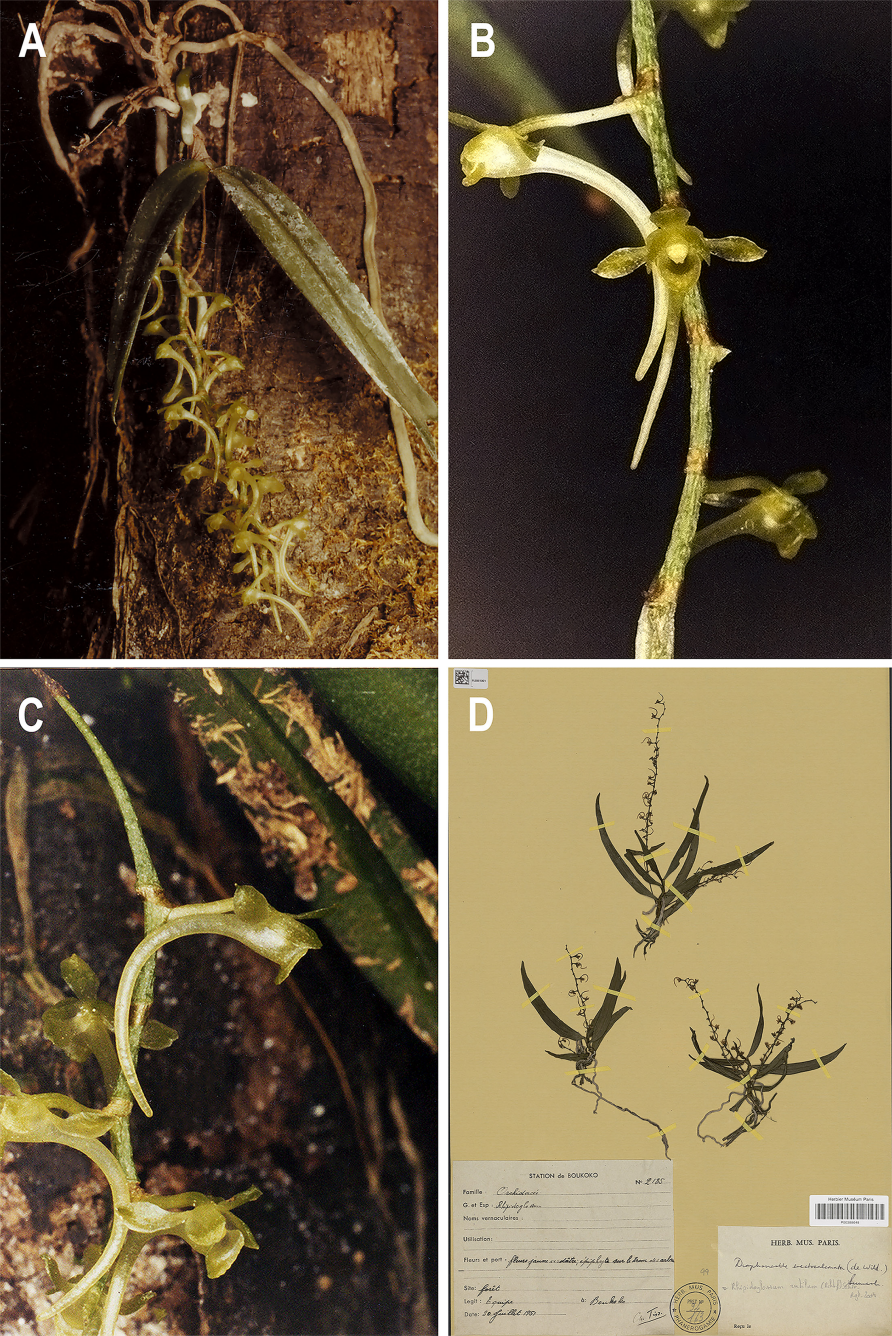
*Rhipidoglossum falcatulum*. **A**. Habit and inflorescence; **B, C**. Flower in frontal (**B**) and lateral (**C**) views; **D**. Holotype *C. Tisserant et al. 2185* (P [P00388648]); **A–C**. Democratic Republic of the Congo, *M. Schaijes 1298* [BR0000025200034, BR0000025200027].

Figs 4, 6, 7, Table 2

#### Type.

**Central African Republic** • Lobaye: Mbaiki, Région de Boukoko, 20 Jul 1951, *C. Tisserant et al. 2185* (holotype P [P00388648!], isotypes P [P00388649!], G [G208985!]).

**Table 2.** Diagnostic traits of *R. falcatulum* sp.nov. and *R. curvatum*, its closest morphologically related species.

	*R. falcatulum* sp.nov.	*R. curvatum*
**Maximum stem size**	60 × 5–9 mm	27 × 2–3 mm
**Leaves**	up to 15, linear-lanceolate to falcate leaves, apex strongly acute, 35–95 × 5–8.5 mm	up to 5, obovate, rarely elliptic, apex acuminate to bilobed, 35–156 × 15–48 mm
**Inflorescence**	16–28-flowered, up to 151 mm long	7–45-flowered, up to 570 mm long
**Flower colour**	perianth pale green, anther cap and column whitish	perianth pale green to yellowish, anther cap and column whitish–yellowish
**Dorsal sepal**	elliptic, 2.3–2.5 × 1–1.2 mm	elliptic to subcircular, 4–4.2 × 3–3.2 mm
**Lateral sepals**	lanceolate, 2.3–2.5 × 1–1.2 mm	oblong-elliptic to slightly obovate, 4–4.3 × 2.5–2.7 mm
**Petals**	elliptic to ovate, 1.2–1.5 × 1–1.2 mm	elliptic to subcircular, 3.1–3.3 × 2.5–2.7 mm
**Lip**	lamina semilunular, minute, convex to slightly concave, ecallose, apex obtuse, 0.7–0.8 × 1.5–1.7 mm	lamina broadly ovate, conspicuous, concave, with a semicircular rim-like callus, apex retuse, 2–2.2 × 4–4.4 mm
**Spur**	10–12 × 0.5–1.7 mm	15–17 × 0.5–0.8 mm
**Column**	1–1.1 × 0.9 mm	1.8–1.9 × 0.9 mm
**Rostellum midlobe**	midlobe oblong, finger-shaped, slightly prominent, apex rounded, 0.46–0.48 × 0.1 mm	midlobe globose, with a middle vertical depression, 1.5–1.55 × 0.4–0.7 mm

#### Diagnosis.

*Rhipidoglossum falcatulum* is similar to *Rhipidoglossum curvatum* but can be distinguished by the following characteristics: stems (up to 15 *vs*. up to 5 leaves); leaf shape (linear to falcate leaves, with a strongly acute apex *vs*. obovate to rarely elliptic, apex acuminate to bilobed); shorter inflorescences (up to 151 mm long *vs*. 570 mm long); and smaller flowers, with a minute semilunular, convex to slightly concave, ecallose lip lamina (vs. conspicuous, broadly ovate, concave lip lamina, with a rim-like semicircular callus) (see Table 2).

#### Description.

Small epiphytic herb, up to 60 mm long. Roots slender, mostly basal and numerous, axillary only at the lower nodes of the stem, one per node, greenish-whitish, 20–61 × 1–2 mm. Stem pendent, slender, unbranched, up to ca. 60 × 1–2 mm, internodes 2.5–5 mm long. Leaves up to 15, distichous, linear-lanceolate to falcate, entire, apex strongly acute, base attenuate, 35–95 × 5–8.5 mm. Inflorescences 1–6, lax, up to 2 per node, axillary, pendent, usually longer than leaves, 16–28-flowered, 48–151 mm long; peduncle glabrous, 11–34 mm long; rachis glabrous, 37–117 mm long; bracts ochreate, 1 × 1 mm, peduncle bracts larger. Flowers small, pale yellow-green, except for greenish white spur. Pedicel and ovary cylindrical, slender, sparsely scaly, 4–5 × 0.5–0.6 mm; dorsal sepal elliptic, apex obtuse, base rounded, entire, 2.3–2.5 × 1–1.2 mm, lateral sepal lanceolate, apex acuminate, base shortly oblong, entire, 2.3–2.5 × 1–1.2 mm; petals elliptic to ovate, apex obtuse, base rounded, entire, 1.2–1.5 × 1–1.2 mm, lip semilunular, apex obtuse, margin entire, convex to slightly concave, ecallose, 0.7–0.8 × 1.5–1.7 mm; spur cylindrical, acute at apex, slightly to strongly curved, 10–12 × 0.5–1.7 mm; column whitish, 1–1.2 mm long; anther cap frontal margin slightly bilobed, with irregularly serrate lobes, whitish-translucent, 0.8–0.9 × 0.8–0.9 mm; stipites two, obclavate, 0.4 mm long, viscidia two, piriform, whitish-translucent; pollinia orbicular, whitish, 0.3 × 0.3 mm; rostellum trilobate, lateral lobes reduced, subtriangular, midlobe oblong, finger-shaped, slightly prominent, apex rounded, shortly longer than the lateral lobes, decurved, 0.46–0.48 × 0.1 mm. Fruit not seen.

#### Distribution.

Central African Republic and Democratic Republic of the Congo, 550–1045 m a.s.l. (Fig. 4). Its small size makes detection difficult, which may partly explain the limited number of specimens and the apparent c. 1,200 km disjunction between known localities in the northern and southern DRC. We therefore expect *R. falcatulum* to be more widely distributed across the lowland Guineo-Congolian forests of Central Africa. Additional botanical surveys are needed to clarify the species distribution range.

#### Habitat and ecology.

*Rhipidoglossum falcatulum* is a rare trunk epiphyte. It was documented in the lowland semi-deciduous Congolian rainforest (approx. 550 m a.s.l.) in the Central African Republic (CAR), in the wetter lowland Congolian rainforest characterised by monodominant patches of *Gilbertiodendron dewevrei* (De Wild.) J. Léonard (approx. at 420 m a.s.l.) in the northern DRC, and in the Central Zambezian miombo woodland, specifically within the *muhulu* vegetation fragments (ca. 1,045 m a.s.l.) in southern DRC. In northern DRC, it was recorded growing on *Isolona hexaloba* (Pierre) Engl. et Diels. In southern DRC, it was recorded growing on *Englerophytum magalismontanum* (Sond.) T.D.Penn. In CAR, it was collected together with *Cyrtorchis brownii* (Rolfe) Schltr., a species typical of seasonally tropical dry forests (Azandi et al. 2016).

#### Phenology.

Flowers from June to late August, and January. Fruits not observed to date.

#### Etymology.

The specific epithet refers to the small falcate leaves, and spur, important diagnostic character of this taxon.

#### Preliminary IUCN conservation assessment.

*Rhipidoglossum falcatulum* is known from seven occurrences based solely on herbarium samples, the most recent collected in 1986. No occurrences were found inside or associated with official protected areas. The seven occurrences represent a total of four biogeographical subpopulations known so far, still believed to exist, considering the presence of native vegetation. They were recorded in the lowland Guineo-Congolian forests close to Boukoko (CAR) and Kisangani (northern DRC), and in dry evergreen forests (“muhulu”) forests, among miombo woodland, close to Kyamasumba (Upper Katanga, DRC). It occurs in four locations with respect to the most serious plausible threat: small-scale agriculture, commercial logging, urban expansion, and slash-and-burn methods for animal husbandry (Blankespoor 1991; Logan and D’Andrea 2012; Burivalova et al. 2015; Delan 2021). Direct human impacts on the vegetation are evident in all locations, including buildings and marked landscape changes for agriculture and animal cultivation. The extent of occurrence (EOO) is calculated as 509,612 km^2^ (being considered as Least Concerned status under criterion B1), whereas its area of occupancy (AOO) is estimated at 16 km^2^ (within the limits for Endangered status under criterion B2), and the number of locations being equal to 3, within the limits for Endangered status under criterion Ba. The projected loss of both subpopulations due to the factors cited above, and contraction of their area of occupancy is associated with a continuing decline in EOO, AOO, habitat extent and quality, and mature individuals. Based on the currently available data, considering our current interpretation of its biology and the potential area of distribution for this taxon, *Rhipidoglossum falcatulum* is assigned a preliminary extinction risk status of **Endangered: EN** B2ab(i, ii, iii, vi, v).

#### Paratypes.

**Central African Republic** • **Lobaye**: Mbaiki, Région de Boukoko, ca. 550 m, 19 Jul 1949, *R.P. Tisserant et al. 1537* (P [P00388650, P00388651], G [G208970]); • *ibid. loc*., 10 Aug 1950, *R.P. Tisserant et al. 2626* (BM [BM000540025], P [P388644, P00388645]). **Democratic Republic of Congo** • **Tshopo**: Banalia, 23 km along road from Kisangani to Bengamisa, 21 Jun 1973, *J. Bokdam 4189* (WAG [WAG.1140141]). • **Lualaba**: Mutshatsha, muhulu de Kyamasumba, 42 km NNW of Kolwezi, 1040 m, 2 Jan 1982, *M. Schaijes 1298* (BR [BR25200010, BR6102008084335-spirit]; • *ibid loc*.; fl. in cult. 1983, *M. Schaijes 1918* (K [K-SPC47374]); • *ibid. loc*., forêt dense sèche sur sable, 1075 m, 20 Jan 1986, *M. Schaijes 2838* (K [K-SPC50377]).

## Discussion

### Taxonomic notes

The description of *R. acuminifolium* and *R. falcatulum* increases the diversity of *Rhipidoglossum* up to 56 species in Tropical Africa and the Gulf of Guinea Islands. A broader phylogenetic framework for the genus, expanding the latest available tree (Farminhão et al. 2021), will be essential to ascertain the exact affinities of the new species and to better understand diversification patterns within one of the largest radiations of epiphytic orchids in the Afrotropics.

Preliminary results from ongoing phylogenetic work on the genus (Macedo et al. in prep.) indicate that, beyond their diagnostic value, column characters, and in particular those of the rostellum, are potentially phylogenetically informative within *Rhipidoglossum*, as previously reported (Farminhão 2016; Macedo et al. 2025). In this context, the morphological similarity between *R. acuminifolium* and *R. delepierreanum* suggests a close affinity between these two taxa, despite a prominent size difference: *R. acuminifolium* exhibits a larger column and rostellum compared to *R. delepierreanum* (see Table 1).

Conversely, the affinity of *R. falcatulum* with *R. curvatum*, supported by overall morphology, is not supported by rostellum morphology. In *R. falcatulum*, we identify one of the smallest columns in the genus (1–1.2 mm long). This miniaturisation is mirrored in the rostellum structure, where the midlobe is significantly reduced (0.46–0.48 mm long), nearly matching the size of the lateral lobes, both of which remain reduced and morphologically undifferentiated. This reduction complicates the interpretation of the rostellum functional role during pollination and its interaction with pollinators. The midlobe itself is oblong and finger-like, bearing little resemblance to that of morphologically allied *R. curvatum*.

*Rhipidoglossum acuminifolium* is morphologically most similar to *R. delepierreanum*, as summarised in Table 1. Both species share a comparable habit, including slender, elongate stems, with markedly unequally bilobed leaves, as well as a similar floral morphology, notably a flabellate, ecallose, lip, beyond the abovementioned differences in column morphology. However, they differ consistently in leaf apex shape, flower size, including spur length. Arguably, these differences could be accommodated at subspecies level, but in the absence of evidence for continuous variation or population-level connectivity, and lacking genetic or phylogeographic data supporting infraspecific differentiation, the use of infraspecific ranks would be premature. Moreover, the increasing tendency to abandon infraspecific ranks in taxonomy (e.g. Burbrink et al. 2022) advises against this option.

### Biogeography

With the description of the two new species, a total of 19 taxa of *Rhipidoglossum* are now recorded in the continental Guineo–Congolian bioregion (as defined by White 1983), representing approximately one third of the genus diversity (African Plant Database 2025). Within Guineo-Congolia, *R. acuminifolium* is restricted to the Cameroon Volcanic Line (CVL). This mountainous region, uplifted during the Miocene (approximately 21 to 7 million years ago; Marzoli et al. 2000; Guillocheau et al. 2015), functions as the region’s most prominent system of “sky islands” and is recognised as an ancient refugial zone and a hotspot of endemism, hosting species with ecological affinities to both lowland and montane rainforests (Nicolas et al. 2011; Missoup et al. 2012; Zimkus and Gvoždík 2013). The distribution of *R. acuminifolium* in the CVL contrasts with that of *R. delepierreanum* in the Western Rift (WR) forests, forming a disjunct pattern between these regions. Such disjunctions have been associated with either long-distance dispersal or habitat discontinuity linked to past climatic fluctuations (Kadu et al. 2011; Couvreur et al. 2021). The connection between the CVL and the WR forests was likely shaped by cycles of forest expansion and contraction during the Late Pleistocene, continuing into the Last Glacial Maximum (Kadu et al. 2011; Couvreur et al. 2021). This pattern of disjunction between the CVL and the WR forests is also encountered in *Rhipidoglossum kamerunense* (Schltr.) Garay (which also occurs in the Katanga Plateau), and several other orchid species. Examples include *Bulbophyllum encephalodes* Summerh., *Habenaria attenuata* Hook.f., *H. peristyloides* A.Rich. (also in the Ethiopian Highlands and Southern Rift Mountains), *Polystachya bifida* Lindl., *P. caloglossa* Rchb.f. (also in Lower Guinea and Katanga Plateau), and *P. retusiloba* Summerh. (African Plant Database 2025; GBIF 2025). These recurrent distribution patterns suggest that this may represent a broader biogeographic signal rather than an isolated case. A broad phylogenetic framework is required to clarify this and many other questions on the diversification of the genus.

The description of *R. falcatulum* also brings elements to a biogeographical discussion. The locality “Muhulu de Kyamasumba”, cited on herbarium labels of *R. falcatulum* from the southern Democratic Republic of the Congo (*M. Schaijes 1298, 1918, 2838*), refers to dense dry forest fragments scattered within the Central Zambezian bioregion (Schmitz 1962; White 1983; Droissart et al. 2018). The muhulus are described as dry semi-deciduous Zambezian formations, with a floristic composition largely derived from Guineo–Congolian elements (Schmitz 1962). They locally represent the climatic vegetation, but due to human fire disturbance, they mostly persist as relictual patches in miombo woodland (Roche 1979). To our knowledge, *R. falcatulum* is among the very few epiphytic orchids recorded in muhulus, together with *Calyptrochilum christyanum* (Rchb.f.) Summerh. and *Polystachya modesta* Rchb.f. (Schmitz 1962). The physiognomy of muhulu appears aligned with drier peripheral semi-evergreen Guineo–Congolian forests, which are also described as transitional formations (White 1983; Droissart et al. 2018). This ecological setting contrasts with the predominantly montane affinities observed in most *Rhipidoglossum* species. Instead, *R. falcatulum* appears to be associated with transitional and marginal forest types. However, considering that it has also been recorded in wetter lowland Guineo–Congolian forest (*J. Bokdam 4189*), its ecological range remains incompletely understood and may partly reflect a limited sampling effort. Further fieldwork across the Guineo–Congolian region will be necessary to clarify this pattern.

### Plant–animal interactions

The documented pollinator record of angraecoid orchids is biased towards sphingophily (Lepidoptera, Sphingidae), and geographically towards the Western Indian Ocean Islands and East and Southern Africa (Farminhão 2021). This is exemplified by the well-known associations with long-tongued hawkmoths (Nilsson et al. 1987; Wasserthal 1997), as well as with short-tongued hawkmoths (Luyt 2002; Martins and Johnson 2007; Houlihan et al. 2019; Azandi et al. 2021). Additional pollinators reported for angraecoids include settling moths (Luyt 2002; Peter and Venter 2017; Farminhão 2021), butterflies (Pailler 2019), raspy crickets (Micheneau et al. 2010), and birds (Micheneau et al. 2008). Our observations of flower visitors in *R. acuminifolium* thus address the vast knowledge gap in non-sphingophilous species, which are estimated to represent ca. 70% of angrecoids (Farminhão 2021). Pollinia removal, multiple head–column contacts, and an apparent morphological fit, in terms of shape and length, between nectar spur and proboscis of nectaring settling moths in *R. acuminifolium*, represent evidence of probable phalaenophily in this species. Previously, pollination by settling moths had only been recorded within angraecoids in *Mystacidium gracile* Harv. (Luyt 2002), *Mystacidium pusillum* Harv. (Luyt 2002; Peter and Venter 2017), and *Rhipidoglossum bilobatum* (Summerh.) Szlach. & Olszewski (Farminhão 2021). In turn, *Rhipidoglossum falcatulum* is also hypothesised here to be primarily pollinated by settling moths based on similarities in flower colour and spur morphology with phalaenophilous *M. pusillum* (Peter & Venter, 2017).

Nectaring of *R. acuminifolium* flowers by ametroidine raspy crickets (cf. *Glomeremus* sp.), which possess mouthpart adaptations for nectar-feeding (Kren et al. 2016), represents only the second documented case of these nocturnal orthopterans acting as flower visitors in Orchidaceae (Micheneau et al. 2010). No florivory was observed in the interactions, similarly to the *Angraecum cadetii* Bosser/*Glomeremus orchidophilus* Hugel et al. 2010 system, from Réunion Island (Micheneau et al. 2010). The apparent mismatch between the orchid column and spur and the cricket mouthparts suggests that these insects are unlikely to be the primary pollinators of *R. acuminifolium*. Nevertheless, they may contribute to incidental pollen transfer between flowers, together with other recorded flower visitors, namely calyptrate flies (Fig. 5). These observations indicate that orthopteran–orchid interactions are not restricted to oceanic island systems, being here reported from the Mount Cameroon sky island. Further research is required to assess how frequently raspy crickets act as flower visitors, and eventually as pollinators, in the Afrotropics and other tropical regions.

## Supplementary Material

XML Treatment for Rhipidoglossum delepierreanum

XML Treatment for Rhipidoglossum acuminifolium

XML Treatment for Rhipidoglossum falcatulum

## Additional information

### Use of AI

No use of AI was reported.

### Author ORCIDs

Arthur Macedo https://orcid.org/0000-0003-1238-493X

Vincent Droissart https://orcid.org/0000-0001-9798-5616

Štěpán Janeček https://orcid.org/0000-0003-1285-6490

Yannick Klomberg https://orcid.org/0000-0002-8566-9728

Marcelo Trovó https://orcid.org/0000-0002-0478-2332

Tariq Stévart https://orcid.org/0000-0002-6212-0361

João Farminhão https://orcid.org/0000-0002-8811-9895

### Data availability

All of the data that support the findings of this study are available in the main text or Supplementary Information.

## Supplementary materials

Supplementary material 1 Additional photographic and unvouchered record of *Rhipidoglossum acuminifolium* made in Bandoumkassa, Cameroon (West Province), 1499 m a.s.l. Data type png Explanation note Record made by Vincent Droissart and his team, and taken for cultivation on 30 November 2021 in Yaoundé Shadehouse, University of Yaoundé I. The plant (cultivation number YA8047) died without producing vouchers. A. Plant habit and size; B. Habit and stem; C. Leaves base; D. Detail of a fruit. https://binary.pensoft.net/file/1607052This dataset is made available under the Open Database License (http://opendatacommons.org/licenses/odbl/1.0/). The Open Database License (ODbL) is a license agreement intended to allow users to freely share, modify, and use this Dataset while maintaining this same freedom for others, provided that the original source and author(s) are credited. Arthur Macedo, Vincent Droissart, Štěpán Janeček, Yannick Klomberg, Marcelo Trovó, Tariq Stévart, João Farminhão

Supplementary material 2 Additional photographic and unvouchered of *Rhipidoglossum delepierreanum* made in Rwanda, Western Province Data type png Explanation note All three records are unvouchered. A–B. Rwanda, Western Province, Gisakura (database record: J. Farminhão and B. Dumbo cult. RWA4); C–D. Rwanda, Western Province (database record: J. Farminhão and B. Dumbo cult. RWA161); E–F. Rwanda, Western Province (database record: J. Farminhão and B. Dumbo cult. RWA162). https://binary.pensoft.net/file/1607053This dataset is made available under the Open Database License (http://opendatacommons.org/licenses/odbl/1.0/). The Open Database License (ODbL) is a license agreement intended to allow users to freely share, modify, and use this Dataset while maintaining this same freedom for others, provided that the original source and author(s) are credited. Arthur Macedo, Vincent Droissart, Štěpán Janeček, Yannick Klomberg, Marcelo Trovó, Tariq Stévart, João Farminhão

Supplementary material 3 Record of *Afroracotis cf. squalida* (Lepidoptera, Erebidae) visiting the flowers of *Rhipidoglossum acuminifolium* and successfully removing the pollinia Data type mov Explanation note This record strongly suggests its role as the probable pollinator. The insect flies carrying the pollinia. This observation was made during fieldwork conducted by Štěpán Janeček and his team on 11 Aug 2017, from 22:12:26 to 22:22:00 WAT, in Mann’s Spring, Mount Cameroon. Although the complete metadata of this record (associated with the orchid identification as *Rhipidoglossum* sp.) was published as supplementary material by Klomberg et al. (2022), Uceda-Gómes et al. (2023), and Sakhalkar et al. (2023), the present file constitutes an unpublished record to date. https://binary.pensoft.net/file/1607054This dataset is made available under the Open Database License (http://opendatacommons.org/licenses/odbl/1.0/). The Open Database License (ODbL) is a license agreement intended to allow users to freely share, modify, and use this Dataset while maintaining this same freedom for others, provided that the original source and author(s) are credited. Arthur Macedo, Vincent Droissart, Štěpán Janeček, Yannick Klomberg, Marcelo Trovó, Tariq Stévart, João Farminhão

Supplementary material 4 Record of *Paschiodes* sp. (Lepidoptera, Erebidae) visiting the flowers of *Rhipidoglossum acuminifolium* Data type mov Explanation note This observation was made during fieldwork conducted by Štěpán Janeček and his team on 19 Aug 2017, from 23:43:27 to 23:43:42 WAT, in Menn’s Spring, Mount Cameroon. Although the complete metadata of this record (associated with the orchid identification as *Rhipidoglossum* sp.) was published as supplementary material by Klomberg et al. (2022), Uceda-Gómes et al. (2023), and Sakhalkar et al. (2023), the present file constitutes an unpublished record to date. https://binary.pensoft.net/file/1607055This dataset is made available under the Open Database License (http://opendatacommons.org/licenses/odbl/1.0/). The Open Database License (ODbL) is a license agreement intended to allow users to freely share, modify, and use this Dataset while maintaining this same freedom for others, provided that the original source and author(s) are credited. Arthur Macedo, Vincent Droissart, Štěpán Janeček, Yannick Klomberg, Marcelo Trovó, Tariq Stévart, João Farminhão

Supplementary material 5 Record of an unidentified species of cricket, cf. *Glomeremus* sp. (Orthoptera, Gryllacrididae, Ametroidini) visiting the flowers of *Rhipidoglossum acuminifolium* Data type mov Explanation note This observation was made during fieldwork conducted by Štěpán Janeček and his team on 11 Aug 2017, from 19:15:07 to 19:17:08 WAT, in Menn’s Spring, Mount Cameroon. Although the complete metadata of this record (associated with the orchid identification as *Rhipidoglossum* sp.) was published as supplementary material by Klomberg et al. (2022), Uceda-Gómes et al. (2023), and Sakhalkar et al. (2023), the present file constitutes an unpublished record to date. https://binary.pensoft.net/file/1607056This dataset is made available under the Open Database License (http://opendatacommons.org/licenses/odbl/1.0/). The Open Database License (ODbL) is a license agreement intended to allow users to freely share, modify, and use this Dataset while maintaining this same freedom for others, provided that the original source and author(s) are credited. Arthur Macedo, Vincent Droissart, Štěpán Janeček, Yannick Klomberg, Marcelo Trovó, Tariq Stévart, João Farminhão

Supplementary material 6 *Zaprionus* sp. (Diptera, Drosophilidae). visiting the flowers and collecting residues of nectar from the spur entrance of *Rhipidoglossum acuminifolium* Data type mov Explanation note This observation was made during fieldwork conducted by Štěpán Janeček and his team on 11 Aug 2017, from 10:48:26 to 10:49:56 WAT, in Menn’s Spring, Mount Cameroon. Although the complete metadata of this record (associated with the orchid identification as *Rhipidoglossum* sp.) was published as supplementary material by Klomberg et al. (2022), Uceda-Gómes et al. (2023), and Sakhalkar et al. (2023), the present file constitutes an unpublished record to date. https://binary.pensoft.net/file/1607057This dataset is made available under the Open Database License (http://opendatacommons.org/licenses/odbl/1.0/). The Open Database License (ODbL) is a license agreement intended to allow users to freely share, modify, and use this Dataset while maintaining this same freedom for others, provided that the original source and author(s) are credited. Arthur Macedo, Vincent Droissart, Štěpán Janeček, Yannick Klomberg, Marcelo Trovó, Tariq Stévart, João Farminhão

Supplementary material 7 *Drosophila* or *Scaptodrosophila* sp. (Diptera, Drosophilidae) visiting the flowers and collecting residues of nectar from the spur entrance and lip surface of *Rhipidoglossum acuminifolium* Data type mov Explanation note This observation was made during fieldwork conducted by Štěpán Janeček and his team on 11 Aug 2017, from 11:27:58 to 11:29:03 WAT, in Menn’s Spring, Mount Cameroon. Although the complete metadata of this record (associated with the orchid identification as *Rhipidoglossum* sp.) was published as supplementary material by Klomberg et al. (2022), Uceda-Gómes et al. (2023), and Sakhalkar et al. (2023), the present file constitutes an unpublished record to date. https://binary.pensoft.net/file/1607058This dataset is made available under the Open Database License (http://opendatacommons.org/licenses/odbl/1.0/). The Open Database License (ODbL) is a license agreement intended to allow users to freely share, modify, and use this Dataset while maintaining this same freedom for others, provided that the original source and author(s) are credited. Arthur Macedo, Vincent Droissart, Štěpán Janeček, Yannick Klomberg, Marcelo Trovó, Tariq Stévart, João Farminhão

Supplementary material 8 Record of an unidentified morphotype of fly described as “orange-whitish-dark fly” (Diptera, Drosophilidae) visiting the flowers of *Rhipidoglossum acuminifolium* Data type mov Explanation note This observation was made during fieldwork conducted by Štěpán Janeček and his team on 11 Aug 2017, from 12:18:46 to 12:19:56 WAT, in Menn’s Spring, Mount Cameroon. Although the complete metadata of this record (associated with the orchid identification as *Rhipidoglossum* sp.) was published as supplementary material by Klomberg et al. (2022), Uceda-Gómes et al. (2023), and Sakhalkar et al. (2023), the present file constitutes an unpublished record to date. https://binary.pensoft.net/file/1607059This dataset is made available under the Open Database License (http://opendatacommons.org/licenses/odbl/1.0/). The Open Database License (ODbL) is a license agreement intended to allow users to freely share, modify, and use this Dataset while maintaining this same freedom for others, provided that the original source and author(s) are credited. Arthur Macedo, Vincent Droissart, Štěpán Janeček, Yannick Klomberg, Marcelo Trovó, Tariq Stévart, João Farminhão

Supplementary material 9 Record of an unidentified species of *Calyptratae* (Diptera, Calyptratae) visiting the flowers of *Rhipidoglossum acuminifolium* Data type mov Explanation note This observation was made during fieldwork conducted by Štěpán Janeček and his team on 11 Aug 2017, from 10:53:02 to 10:57:22 WAT, in Menn’s Spring, Mount Cameroon. Although the complete metadata of this record (associated with the orchid identification as *Rhipidoglossum* sp.) was published as supplementary material by Klomberg et al. (2022), Uceda-Gómes et al. (2023), and Sakhalkar et al. (2023), the present file constitutes an unpublished record to date. https://binary.pensoft.net/file/1607060This dataset is made available under the Open Database License (http://opendatacommons.org/licenses/odbl/1.0/). The Open Database License (ODbL) is a license agreement intended to allow users to freely share, modify, and use this Dataset while maintaining this same freedom for others, provided that the original source and author(s) are credited. Arthur Macedo, Vincent Droissart, Štěpán Janeček, Yannick Klomberg, Marcelo Trovó, Tariq Stévart, João Farminhão

Supplementary material 10 Unidentified species of wasp (Hymenoptera) visiting the flowers, and (apparently) feeding on nectar residues on the flower surface of *Rhipidoglossum acuminifolium* Data type mov Explanation note This observation was made during fieldwork conducted by Štěpán Janeček and his team on 11 Aug 2017, from 11:41:16 to 11:41:27 WAT, in Menn’s Spring, Mount Cameroon. Although the complete metadata of this record (associated with the orchid identification as *Rhipidoglossum* sp.) was published as supplementary material by Klomberg et al. (2022), Uceda-Gómes et al. (2023), and Sakhalkar et al. (2023), the present file constitutes an unpublished record to date. https://binary.pensoft.net/file/1607061This dataset is made available under the Open Database License (http://opendatacommons.org/licenses/odbl/1.0/). The Open Database License (ODbL) is a license agreement intended to allow users to freely share, modify, and use this Dataset while maintaining this same freedom for others, provided that the original source and author(s) are credited. Arthur Macedo, Vincent Droissart, Štěpán Janeček, Yannick Klomberg, Marcelo Trovó, Tariq Stévart, João Farminhão

Supplementary material 11 Record of *Cinnyris reichenowi* (Nectariniidae) visiting and thieving nectar from *Rhipidoglossum acuminifolium* Data type mov Explanation note This observation was made during fieldwork conducted by Štěpán Janeček and his team on 11 Aug 2017, from 13:32:35 to 13:32:44 WAT, in Menn’s Spring, Mount Cameroon. Although the complete metadata of this record (associated with the orchid identification as *Rhipidoglossum* sp.) was published as supplementary material by Klomberg et al. (2022), Uceda-Gómes et al. (2023), and Sakhalkar et al. (2023), the present file constitutes an unpublished record to date. https://binary.pensoft.net/file/1607062This dataset is made available under the Open Database License (http://opendatacommons.org/licenses/odbl/1.0/). The Open Database License (ODbL) is a license agreement intended to allow users to freely share, modify, and use this Dataset while maintaining this same freedom for others, provided that the original source and author(s) are credited. Arthur Macedo, Vincent Droissart, Štěpán Janeček, Yannick Klomberg, Marcelo Trovó, Tariq Stévart, João Farminhão

## References

[B1] African Plant Database [v. 4.0.0] (2025) Conservatoire et Jardin botaniques de la Ville de Genève and South African National Biodiversity Institute, Pretoria. http://africanplantdatabase.ch [accessed 04.11.2025]

[B2] Azandi L, Stevart T, Sonké B, Simo-Droissart M, Avsns ML, Droissart V (2016) Synoptic revision of the genus *Cyrtorchis* Schltr. (Angraecinae, Orchidaceae) in Central Africa, with the description of a new species restricted to submontane vegetation. Phytotaxa 267(3): 165–186. 10.11646/phytotaxa.267.3.1

[B3] Azandi L, Stevart T, Sonké B, Simo-Droissart M, D'haijère T, Droissart V (2021) Taxonomic description and pollination ecology of *Cyrtorchis okuensis* (Orchidaceae, Angraecinae), a new species endemic to the Cameroon Volcanic Line. Plant Ecology and Evolution 154(3): 487–496. 10.5091/plecevo.2021.1823

[B4] Bachman S, Moat J, Hill AW, de la Torre J, Scott B (2011) Supporting Red List threat assessments with GeoCAT: geospatial conservation assessment tool. ZooKeys 150: 117–126. 10.3897/zookeys.150.2109 PMC3234434 22207809

[B5] Beentje H (2016) The Kew Plant Glossary, an Illustrated Dictionary of Plant Terms. Kew Publishing, Royal Botanic Gardens, Kew, Richmond, 160 pp.

[B6] Blankespoor GW (1991) Slash-and-burn shifting agriculture and bird communities in Liberia, West Africa. Biological Conservation 57: 41–71. 10.1016/0006-3207(91)90107-k

[B7] Burbrink FT, Crother BI, Murray CM, Smith BT, Ruane S, Myers EA, Pyron RA (2022) Empirical and philosophical problems with the subspecies rank. Ecology and Evolution 12(7): e9069. 10.1002/ece3.9069 PMC9271888 35845367

[B8] Burivalova Z, Lee TM, Giam X, Seekercioglu CH, Wilcove DS, Koh LP (2015) Avian responses to selective logging shaped by species traits and logging practices. Proceedings of the Royal Society B 282: e20150164. 10.1098/rspb.2015.0164 PMC4455798 25994673

[B9] Couvreur TLP, Dauby G, Blach-Overgaard A, Deblauwe V, Dessein S, Droissart V, Hardy OJ, Harris DJ, Janssens SB, Ley AC, Mackinder BA, Sonké B, Sosef MSM, Stévart T, Svenning J-C, Wieringa JJ, Faye A, Missoup AD, Tolley KA, Nicolas V, Ntie S, Fluteau F, Robin C, Guillocheau F, Barboni D, Sepulchre P (2021) Tectonics, climate and the diversification of the tropical African terrestrial flora and fauna. Biological reviews 96(1): 16–51. 10.1111/brv.12644 PMC7821006 32924323

[B10] Cribb PJ, Fay JM (1987) Orchids of the Central African Republic: A provisional checklist. Kew Bulletin 42(3): 711–737. 10.2307/4110085

[B11] Cribb PJ (1989) Orchidaceae (part 3). In: Polhill RM (Ed.) Flora of Tropical East Africa. Balkema, Rotterdam, 413–652. 10.1201/9780203755815

[B12] Cribb PJ, Hemp A (2022) *Rhipidoglossum pareense* (Orchidaceae: Epidendroideae), a new species from Tanzania. Kew Bulletin 77: 685–689. 10.1007/S12225-022-10027-2

[B13] Delan WRJ (2021) A review of the conservation status of birds in the Guineo-Congolian forest of Africa. Journal of Field Ornithology 92(4): 342–364. 10.1111/jofo.12388

[B14] D’haijère T, Kaymak E, Boom AF, Hardy OJ, Stévart T, Mardulyn P (2022) Diversification of the orchid genus *Tridactyle*: Origin of endemism on the oceanic islands of São Tomé & Príncipe in the Gulf of Guinea. Journal of Biogeography 49(3): 523–536. https://onlinelibrary.wiley.com/doi/10.1111/jbi.14324

[B15] Droissart V (2009) Etude taxonomique et biogéographique des plantes endémiques d’Afrique centrale atlantique: le cas des Orchidaceae. Ph. D. Thesis, Université Libre de Bruxelles, Belgium, 270 pp.

[B16] Droissart V, Dauby G, Hardy OJ, Deblauwe V, Harris DJ, Janssens S, Mackinder BA, Blach-Overgaard A, Sonké B, Sosef MSM, Stévart T, Svenning J-C, Wieringa JJ, Couvreur TLP (2018) Beyond trees: Biogeographical regionalization of tropical Africa. Journal of Biogeography 45: 1153–1167. 10.1111/jbi.13190

[B17] Farminhão JNM (2016) To lump or not to lump: revisiting the taxonomy of *Rhipidoglossum* and its allied genera *Cribbia*, *Margelliantha*, and *Rhaesteria* (Angraecinae, Orchidaceae). MSc Thesis, Université libre de Bruxelles, 67 pp.

[B18] Farminhão JNM, Meerts P, Descourvières P, Droissart V, Simo-Droissart M, Stévart T (2018) A revised concept of *Rhipidoglossum* (Angraecinae, Orchidaceae). Phytotaxa 349: 247–256. 10.11646/phytotaxa.349.3.5

[B19] Farminhão JNM (2021) Advances in angraecoid orchid systematics in Tropical Africa and Madagascar: new taxa and hypotheses for their diversification. PhD Thesis, Université libre de Bruxelles, 364 pp.

[B20] Farminhão JNM, Cribb P (2020) Two new species of *Rhipidoglossum* (Orchidaceae: Angraecinae) from Tanzania and Zimbabwe. Kew Bulletin 75(30): 1–8. 10.1007/S12225-020-09888-2

[B21] Fischer E, Killmann D, Delepierre G, Lebel JP (2010) The Orchids of Rwanda. An illustrated Field Guide. Universität Koblenz-Landau, Koblenz, 439 pp.

[B22] Fischer E, Killmann D, Lebel JP, Delepierre G (2011) Neue Kombinationen in der Gattung *Rhipidoglossum* – New combination in the genus *Rhipidoglossum*. Die Orchidee 62(6): e445.

[B23] GBIF [Global Biodiversity Information Facility] (2025) GBIF Occurrence Download. 10.15468/dl.4frxtp [accessed 01.11.2025]

[B24] Geerinck D, Delepierre G, Lebel JP (1998) Supplément à l’étude des Orchidaceae du Rwanda (II) Belgian Journal of Botany 130(2): 135–138.

[B25] Geerinck D (1992) Orchidaceae (seconde partie). In: Bamps P (Ed.) Flore d’Afrique Centrale (Zaïre–Rwanda–Burundi) spermatophytes. Jardin Botanique National de Belgique, Leuven, 297–780. 10.2307/3668313

[B26] Google (2025) Google Earth Pro software. Version 7.3.6.9. https://earth.google.com/web [accessed 10.02.2025]

[B27] Guillocheau F, Chelalou R, Linol B, Dauteuil O, Robin C, Mvondo F, Callec Y, Colin JP (2015) Cenozoic landscape evolution in and around the Congo Basin: Constraints from sediments and planation surfaces. In: de Wit MJ, Guillocheau F, de Wit MCJ (Eds) Geology and Resource Potential of the Congo Basin, Regional Geology Reviews. Springer-Verlag, Berlin and Heidelberg, 271–313. 10.1007/978-3-642-29482-2_14

[B28] Houlihan PR, Stone M, Clem SE, Owen M, Emmel TC (2019) Pollination ecology of the ghost orchid (*Dendrophylax lindenii*): A first description with new hypotheses for Darwin’s orchids. Scientific Reports 9(1): e12850. 10.1038/s41598-019-49387-4 PMC6731287 31492938

[B29] IUCN (2024) Guidelines for using the IUCN Red List categories and criteria, version 16. IUCN Standards and Petitions Subcommittee. https://nc.iucnredlist.org/redlist/content/attachment_files/RedListGuidelines.pdf [accessed 1.11.2025]

[B30] Janeček S, Chmel K, Mlíkovský J, Uceda-Gómez G, Janečková P, Fominka NT, Njie MM, Ewome FL (2022) Spatiotemporal pattern of specialization of sunbird-plant networks on Mt. Cameroon. Oecologia 199: 885–896. 10.1007/s00442-022-05234-4 35947185

[B31] Janeček Š, Uceda-Gómez G, Janečková P, Tropek R, Fominka NT, Njie MM, Mlíkovský J, Kamga SM, Molua LL, Ewome FL (2024) Food resource partitioning between males and females of Volcano Sunbird (*Cinnyris preussi*) on Mount Cameroon. Journal of Ornithology 165: 1025–1038. 10.1007/s10336-024-02187-8

[B32] Kadu CA, Schueler S, Konrad H, Muluvi GM, Eyog-Matig O, Muchugi A, Williams VL, Ramamonjisoa L, Kapinga C, Foahom B, Katsvanga C, Hafashimana D, Obama C, Geburek T (2011) Phylogeography of the Afromontane *Prunus africana* reveals a former migration corridor between East and West African highlands. Molecular Ecology: 165–178. 10.1111/j.1365-294X.2010.04931.x 21087325

[B33] Klomberg Y, Tropek R, Mertens JEJ, Kobe IN, Hodeček J, Raška J, Fominka NT, Souto-Vilarós D, Janečková P, Janeček Š (2022) Spatiotemporal variation in the role of floral traits in shaping tropical plant-pollinator interactions. Ecology Letters 25(4): 839–850. 10.1111/ele.13958 35006639

[B34] Kren HW, Fournel J, Bauder JA, Hugel S (2016) Mouthparts and nectar feeding of the flower visiting cricket *Glomeremus orchidophilus* (Gryllacrididae). Arthropod Structure & Development 45(3): 221–229. 10.1016/j.asd.2016.03.002 27067454

[B35] Lézine AM, Achoundong G, Tchiengué B (2025) Vulnerability of montane forests of Cameroon in the face of climate change: insight from the last glacial-interglacial transition. Quaternary Science Reviews 363(1): e109410. 10.1016/j.quascirev.2025.109410

[B36] Logan AL, D’Andrea AC (2012) Oil palm, arboriculture, and changing subsistence practices during Kintampo times (3600–3200 BP, Ghana). Quaternary International 249(6): 63–71. 10.1016/j.quaint.2010.12.004

[B37] Luyt RP (2002) Pollination and evolution of the genus *Mystacidium* (Orchidaceae). MSc Thesis, University of Natal, 90 pp. http://hdl.handle.net/10413/10209

[B38] Marzoli A, Piccirillo EM, Renne PR, Bellieni G, Iacumin M, Nyobe JB, Tongwa AT (2000) The Cameroon Volcanic Line revisited: Petrogenesis of continental basaltic magmas from lithospheric and asthenospheric mantle sources. Journal of Petrology 41(1): 87–109. 10.1093/petrology/41.1.87

[B39] Macedo A, Trovó M, Stévart T, Farminhão J (2025) A new species of *Rhipidoglossum* (Orchidaceae, Angraecinae) from the Western Rift Valley (Africa). Plant Ecology and Evolution 158(2): 248–259. 10.5091/plecevo.155517

[B40] Martins DJ, Johnson SD (2007) Hawkmoth pollination of aerangoid orchids in Kenya, with special reference to nectar sugar concentration gradients in the floral spurs. American Journal of Botany 94(4): 650–659. 10.3732/ajb.94.4.650 21636433

[B41] Micheneau C, Fournel J, Humeau L, Pailler T (2008) Orchid–bird interactions: a case study from Angraecum (Vandeae, Angraecinae) and *Zosterops* (white-eyes, Zosteropidae) on Reunion Island. Botany 86(10): 1143–1151. 10.1139/B08-068

[B42] Micheneau C, Fournel J, Warren BH, Hugel S, Gauvin-Bialecki A, Pailler T, Strasberg D, Chase MW (2010) Orthoptera, a new order of pollinator. Annals of Botany 105(3): 355–364. 10.1093/aob/mcp299 PMC2826249 20067913

[B43] Missoup AD, Nicolas V, Wendelen W, Keming E, Bilong Bilong CF, Couloux A, Atanga E, Hutterer R, Denys C (2012) Systematics and diversification of *Praomys* species (Rodentia: Muridae) endemic to the Cameroon Volcanic Line (West Central Africa). Zoologica Scripta 41: 327–345. 10.1111/j.1463-6409.2012.00541.x

[B44] Nicolas V, Missoup AD, Denys C, Bryja J, Kerbis Peterhans J, Katuala P, Couloux A, Colyn M (2011) The role of rivers and Pleistocene Forest refuges in shaping genetic diversity in *Praomys misonnei* in tropical Africa. Journal of Biogeography 38: 191–207. 10.1111/j.1365-2699.2010.02399.x

[B45] Nilsson LA, Johnsson L, Ralison L, Randrianjohany E (1987) Angraecoid orchids and hawkmoths in central Madagascar: specialized pollination systems and generalist foragers. Biotropica 19(4): 310–318. 10.2307/2388628

[B46] Nodza GI, Onuminya TO, Ogundipe OT (2022) Preliminary conservation checklist of orchid of Gashaka Gumti National Park, Nigeria. Journal of Tropical Biology & Conservation (JTBC) 19: 29–46. 10.51200/jtbc.v19i.3936

[B47] Pailler T (2019) First report of *Papilio* pollination in angraecoid orchids. Abstract Booklet – the 7^th^ International Orchid Conservation Congress, 28 May–1 Jun 2019. Royal Botanic Gardens, Kew, United Kingdom.

[B48] Peter CI, Venter N (2017) Generalist, settling moth pollination in the endemic South African twig epiphyte, *Mystacidium pusillum* Harv. (Orchidaceae). Flora 232: 16–21. 10.1016/j.flora.2016.11.014

[B49] QGIS Development Team (2024) QGIS Geographic Information System. Version 3.40. OpenSource Geospatial Foundation Project. https://qgis.org [accessed 10.02.2025]

[B50] Radford AE, Dickison WC, Massey JR, Bell CR (1974) Vascular Plant Systematics. Harper & Row Publishers, New York, 891 pp.

[B51] Roche E (1979) Végétation ancienne et actuelle de l’Afrique Centrale. African Economic History 7: 30–37. 10.2307/3601201

[B52] Sanford WW (1971) The orchid flora of equatorial Guinea in relation to that of west Africa. Mitteilungen der Botanischen Staatssammlung München 10: 287–298.

[B53] Sakhalkar SP, Janeček Š, Klomberg Y, Mertens JEJ, Hodeček J, Tropek R (2023) Cheaters among pollinators: Nectar robbing and thieving vary spatiotemporally with floral traits in Afrotropical forests. Ecosphere 14(11): e4696. 10.1002/ecs2.4696

[B54] Schmitz A (1962) Les muhulu du Haut-Katanga méridional. Bulletin du Jardin botanique de l’État à Bruxelles 32(3): 221–299. 10.2307/3667284

[B55] Solefack MCM, Chabrerie O, Gallet-Moron E, Nkongmeneck BA, Leumbe ONL, Decocq G (2012) Analysing deforestation by remote sensing coupled with structural equation models: example of the cloud forest of Mount Oku (Cameroon). Acta Botanica Gallica 159(4): 451–466. 10.1080/12538078.2012.750583

[B56] Stévart T, Akouangou E, Andriamahefarivo L, Andriatsiferana F, Azandi L, Bakita B, Biteau J-P, D’haijère T, Farminhão JNM, Kamdem G, Lowry II PP, Mayogo S, Nyangala C, Oliveira F, Rajaonarivelo N, Rakotoarivony F, Ramandimbisoa B, Randrianasolo A, Razafindramanana J, Razanatsima AA, Simo-Droissart M, Sonké B, Verlynde S, Williams T, Droissart V (2020) The Missouri botanical garden, Africa and Madagascar. In: Hermans J, Hermans C, Linsky J, Li CW (Eds) World Orchid Collections 2020. Taiwan Orchid Growers Association (TOGA), Taiwan, 26–43.

[B57] Summerhayes VS (1937) A review of the genus *Rhipidoglossum* Schltdl. Blumea, Supplement 1: 78–86.

[B58] Summerhayes VS (1968) Orchidaceae. In: Hutchinson J, Dalziel JM (Eds) Flora of West Tropical Africa (2^nd^ edn.). 3. Calendula Horticultural Books, Londen, 181–276.

[B59] Szlachetko DL, Olszewski TS (2001) Orchidacées 3. In: Achoundong G, Morat P (Eds) Flore du Cameroun 36. Ministère de la Recherche Scientifique et Technique, Yaoundé, 868 pp.

[B60] Szlachetko DL, Grochcka E, Baranow P, Norwak S, Mytnik J, Oledryzynska O, Rutkowski P (2021) Orchidaceae of West-Central Africa (Vol. 3): Vandoideae (continued). Koeltz Scientific Books, Koenigsheim, Germany, 555 pp.

[B61] Thiers B (2025) Index Herbariorum: a global directory of public herbaria and associated staff. New York Botanical Garden’s Virtual Herbarium, New York. https://sweetgum.nybg.org/science/ih/ [accessed 10.02.2025]

[B62] Teisher J, Stimmel H (2026) Tropicos MO Specimen Data. Missouri Botanical Garden. Occurrence dataset. 10.15468/hja69f [accessed via GBIF.org 23.03.2026]

[B63] Tisserant C (1950) Catalogue de la Flore d’Oubangi-Chari. Orchidaches. Mémoires de l’Institut d’études centrafricaines N° 2: 80–82.

[B64] Uceda-Gómez G, Chmel K, Janečková P, Mlíkovský J, Klomberg Y, Ewome FL, Molua LL, Njie MM, Tropek R, Janeček Š (2023) Drivers of sunbird-plant interactions on Mount Cameroon: Between neutrality and niche-based processes. Biotropica 56(1): 136–148. 10.1111/btp.13290

[B65] Wasserthal LT (1997) The pollinators of the Malagasy star orchids *Angraecum sesquipedale*, *A. sororium* and *A. compactum* and the evolution of extremely long spurs by pollinator shift. Botanica Acta 110(5): 343–359. 10.1111/j.1438-8677.1997.tb00650.x

[B66] White F (1983) The vegetation of Africa: a descriptive memoir to accompany the UNESCO/AETFAT/UNSO vegetation map of Africa. UNESCO, Paris. 10.5281/zenodo.13425411

[B67] Wilson MR, Carapezza A, Deeming J, Hosseini R, Kment P, Linnavuori P, Štys P (2017) Rauno Linnavuori: Entomologist and explorer. Entomologica Americana 122(4): 505–512. 10.1664/1947-5144-122.4.505

[B68] Zimkus BM, Gvoždík V (2013) Sky Islands of the Cameroon Volcanic Line: A diversification hot spot for puddle frogs (Phrynobatrachidae: *Phrynobatrachus*). Zoologica Scripta 42: 591–611. 10.1111/zsc.12029

