# Supplementary figures and images for: Two new species of Rhipidoglossum (Orchidaceae, Angraecinae) from Central Africa, probably pollinated by settling moths
Source: PhytoKeys. 2026 Apr 24;274:1–30. doi: 10.3897/phytokeys.274.184429 (PMC13135204; doi:10.3897/phytokeys.274.184429)

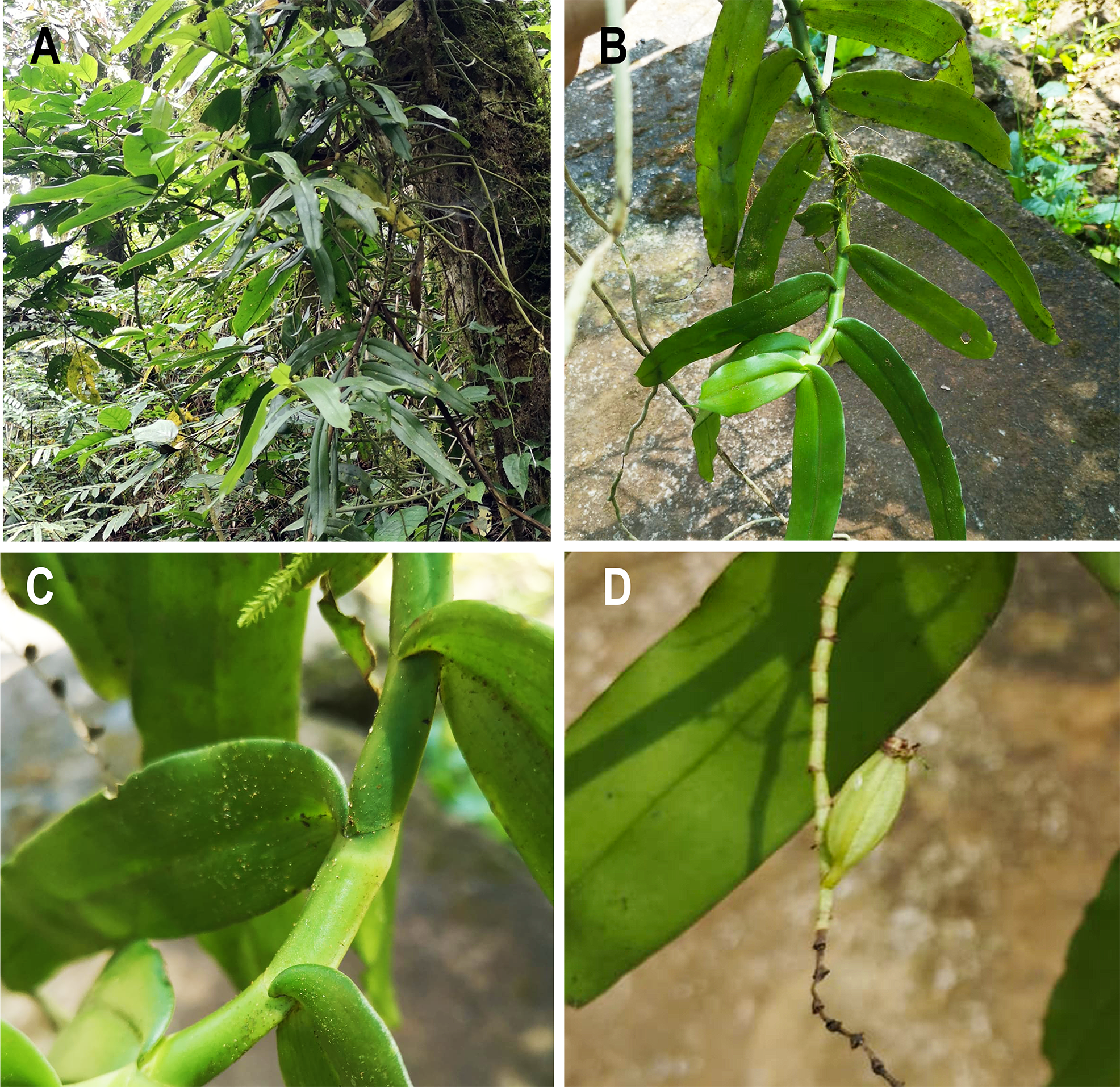

Supplement: Supplementary material 1 — Additional photographic and unvouchered record of Rhipidoglossum acuminifolium made in Bandoumkassa, Cameroon (West Province), 1499 m a.s.l. [file phytokeys-274-001_article-184429__-s001.png]

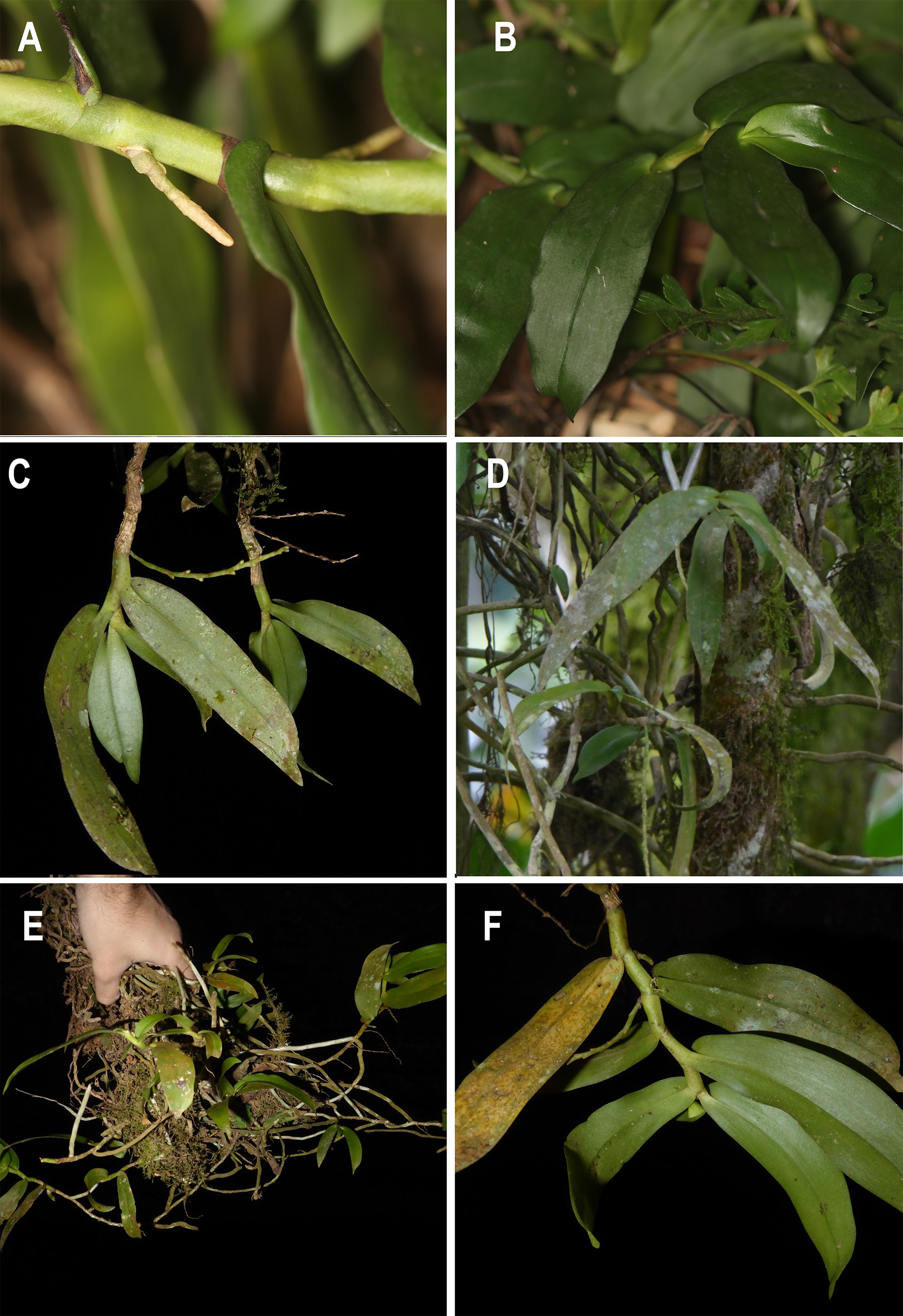

Supplement: Supplementary material 2 — Additional photographic and unvouchered of Rhipidoglossum delepierreanum made in Rwanda, Western Province [file phytokeys-274-001_article-184429__-s002.png]
